# Depolarizing GABA/glycine synaptic events switch from excitation to inhibition during frequency increases

**DOI:** 10.1038/srep21753

**Published:** 2016-02-25

**Authors:** Pascal Branchereau, Daniel Cattaert, Alain Delpy, Anne-Emilie Allain, Elodie Martin, Pierre Meyrand

**Affiliations:** 1 Univ. Bordeaux, INCIA, UMR 5287, F-33615 Pessac, France; 2 CNRS, INCIA, UMR 5287, F-33615 Pessac, France

## Abstract

By acting on their ionotropic chloride channel receptors, GABA and glycine represent the major inhibitory transmitters of the central nervous system. Nevertheless, in various brain structures, depolarizing GABAergic/glycinergic postsynaptic potentials (dGPSPs) lead to dual inhibitory (shunting) and excitatory components, the functional consequences of which remain poorly acknowledged. Indeed, the extent to which each component prevails during dGPSP is unclear. Understanding the mechanisms predicting the dGPSP outcome on neural network activity is therefore a major issue in neurobiology. By combining electrophysiological recordings of spinal embryonic mouse motoneurons and modelling study, we demonstrate that increasing the chloride conductance (g_Cl_) favors inhibition either during a single dGPSP or during trains in which g_Cl_ summates. Finally, based on this summation mechanism, the excitatory effect of EPSPs is overcome by dGPSPs in a frequency-dependent manner. These results reveal an important mechanism by which dGPSPs protect against the overexcitation of neural excitatory circuits.

By acting on their ionotropic chloride channel receptor, GABA and glycine represent the major inhibitory amino acid transmitters of the central nervous system (CNS). These inhibitory transmitters control the input-output (I-O) relationship of excitatory drives impinging on neurons. However, under numerous circumstances, GABAergic/glycinergic postsynaptic potentials are depolarizing (dGPSPs) and exert mixed excitatory (depolarizing) and inhibitory (shunting) effects on the I-O relationship^1^,^2^,^3^,^4^,^5^,^6^,^7^. From a functional perspective, dGPSPs control numerous crucial neuronal processes, such as spontaneous activities in the developing CNS^8^,^9^ and tuning and gain-setting mechanisms^1^,^10^. The effects of dGPSPs on the I-O relationship depend on the following major parameters: the driving force for chloride ions^11^,^12^, the peak chloride conductance g_Clp_, the neuronal input resistance (R_in_) and the spatial arrangement of these synapses^13^. The inhibitory effect of dGPSPs on excitatory glutamatergic inputs largely depends on their relative timing^14^ and location on the target neuron^1^,^13^. The effect of dGPSPs is also controlled by other mechanisms, such as HCO3^−^ permeability of the GABA_A_/glycine receptors, changes in [K^+^]_o_ after massive synaptic activation of GABA_A_R^15^, and local differences in E_Cl_ values in somatic, axonal and dendritic compartments^16^. The relationship between the inhibitory and excitatory components of dGPSPs is therefore a complex phenomenon.

Here, we address the relative roles of the excitatory (depolarization) and inhibitory (shunting) components of dGPSPs by a quantitative analysis of their interplay. We also consider the interaction of three parameters (E_Cl_, g_Clp_ and E_Rest_) and their modulation by neuron passive properties.

The present study addresses this question 1) by precisely assessing the magnitude and time course of the inhibitory and excitatory effects of a single dGPSP; 2) by analyzing the functional outcome of repetitive dGPSPs as a function of frequency; and 3) by considering their interaction with excitatory drives, which occurs in most neuronal types. For this purpose, a neuron that naturally exhibited various E_Cl_ values and shapes (*i.e*., size and R_in_) was required. Therefore, we examined mouse spinal motoneurons (MNs), which express these parameter alterations during the course of development^17^. Spinal MNs undergo dramatic morphological changes during development, and their dendritic tree largely evolves to integrate numerous excitatory and inhibitory synaptic inputs. Moreover, these neurons exhibit dramatic changes in E_Cl_ during development. We therefore used whole-cell patch-clamp recordings from immature E13.5 and more mature E17.5 spinal MNs, in which [Cl^−^]_i_ is high and low, respectively^17^. The effect of g_Clp_ was probed by controlled local applications of a GABA_A_R agonist (*via* pressure microejection).

To gain a deeper understanding of the mechanisms controlling dGPSP effects, we combined a physiological analysis with computational simulations. These simulations allowed us to measure and model the role of each parameter (E_m_, E_Cl_, g_Clp_, neuron size and the input resistance R_in_) in the magnitude and time course of inhibition and excitation during single dGPSPs.

This study demonstrates that regardless of the neuron passive properties, increasing g_Clp_ may result in a switch from excitatory to inhibitory dGPSP depending on the E_Cl_. We also show that dGPSP trains can be excitatory at low rates and inhibitory at high rates. Finally, we demonstrate that dGPSP trains modulate the outcome of excitatory drives (EPSPs) as follows: 1) time-locked EPSPs that are generated during the early phase of dGPSPs are inhibited and time-locked EPSPs that are generated during the late phase of dGPSPs are facilitated; 2) the transition from excitation to inhibition is more abrupt as g_Clp_ increases; and 3) random EPSP barrages are facilitated by low-rate dGPSP trains and inhibited by high-rate dGPSP trains, with the inhibition becoming more abrupt as g_Clp_ increases. These results indicate that compared with classical EPSPs, excitatory dGPSPs switch to potent inhibition as their discharge frequency increases, which precludes the overexcitation of the neuronal network and represents a fundamental property of these dGPSPs.

## Results

To understand the mechanisms governing the balance between the excitatory and inhibitory effects of dGPSPs on the neuronal output, we performed patch-clamp recordings from neurons of different shapes and sizes, as well as realistic simulations. These approaches allowed us to estimate the influence of neuron size and its passive properties, including the GABA/glycine-induced conductance increase, the chloride equilibrium and the resting membrane potential on the dGPSP outcome. Additionally, simulations were performed to explore the functional consequence of dGPSP frequency on the neuron output. Although an axo-somato-dendritic gradient may occur in spinal MNs in which the E_GABA_ values from the AIS to the soma and dendrites become progressively more negative, which was demonstrated in postnatal hippocampal and cortical pyramidal neurons^16^, we only analyzed dGPSPs produced as the somatic GABAergic receptors were activated. All of the dGPSPs illustrated in the present study were attributed to fast synaptic GABA/glycine events, thus implying the presence of chloride ions, the intracellular concentration of which is primarily controlled by the cation-chloride co-transporters Na^+^/K^+^/2Cl^−^ type 1 (NKCC1) and K^+^/Cl^−^ type 2 (KCC2), with NKCC1 and KCC2 acting as the intracellular chloride accumulator and extruder, respectively^18^,^19^. Long-lasting GABA-mediated depolarizations, which are induced with massive GABA_A_R activation and involve an increase in [K^+^]_o_ and HCO3^−^ ions^20^,^21^, were not addressed in our study.

### Morphological and electrical passive properties of E13.5 and E17.5 MNs

**Figure 1:**
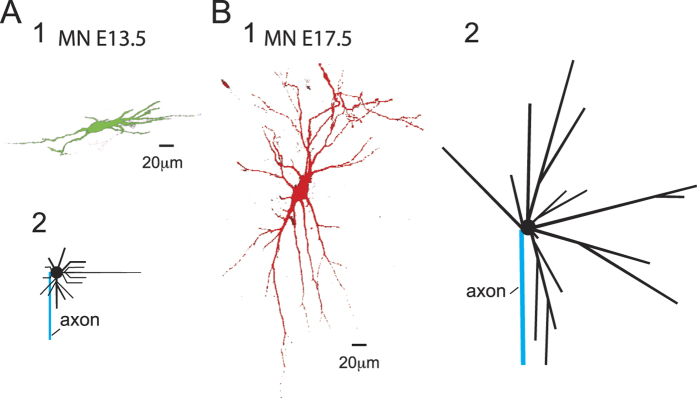
Morphology of E13.5 and E17.5 MNs and their canonical counterparts that were used in the simulations. (**A**_**1**_) Photograph of a neurobiotin-filled E13.5 MN as revealed with fluorescent dye. (**A**_**2**_) Morphology of the canonical E13.5 MN that was used in the simulation. (**B**_**1-2**_) The same disposition as in (**A**_**1-2**_) but for E17.5 MNs.

During development, spinal MNs undergo dramatic changes in size and passive properties^17^. For our purposes, we used spinal MNs at E13.5 and E17.5 because their passive properties and morphologies were clearly different. Indeed, in immature E13.5 embryos, MNs exhibited a small cell body (diameter = 12.1 ± 0.3 μm, n = 4, Fig. 1A1) and an R_in_ of 887 ± 55 MΩ (n = 12). In contrast, E17.5 MNs dramatically matured; their cell body diameter increased to 17.2 ± 0.2 μm (n = 3) as did their dendritic tree (compare Fig. 1A1,1B1) (see also Suppl. Tables 1–2 in the Supplemental Information), and their R_in_ decreased to 145 ± 24 MΩ (n = 13). The R_in_ of the E13.5 MNs was approximately 6 times higher than that of the E17.5 MNs, and both exhibited dGPSPs. The canonical E13.5 and E17.5 neurons were constructed from the morphological and electrical mean properties of real MNs (Fig. 1A2,1B2). The first step was to monitor the effects of dGPSPs on these two neurons.

### Depolarizing GPSPs are purely excitatory at E13.5 and exclusively inhibitory at E17.5

**Figure 2:**
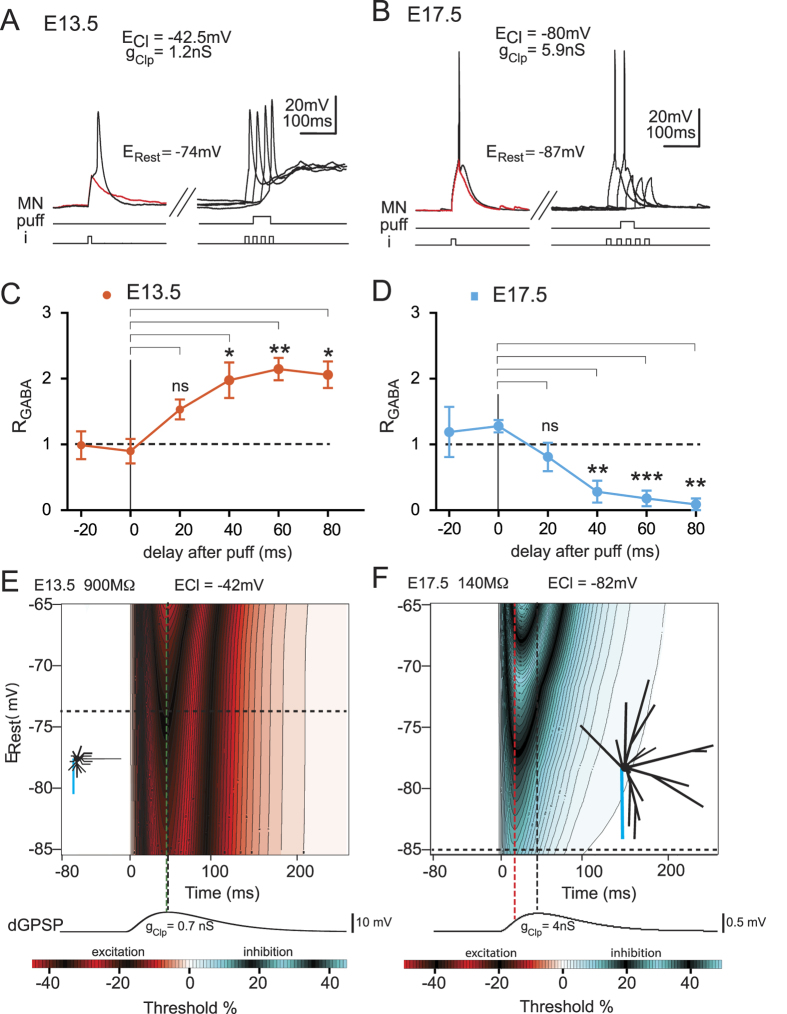
Time course of the excitatory and inhibitory components of dGPSPs in E13.5 and E17.5 neurons with physiological E_Cl_ values. (**A**) Patch-clamp recordings of E13.5 MN responses (MN) to a threshold current pulse (i) in the absence (left traces) and presence (right traces) of an isoguvacine-induced response (puff). The red trace is an example of a spike triggering failure during the four-pulse series prior to the isoguvacine puff (see Suppl. Fig. 3). (**B**) The same disposition as in (**A**) but for an E17.5 MN; *E*_*Cl*_: reversal potential for chloride; *g*_*Clp*_: peak conductance; *E*_*Rest*_: resting membrane potential. (**C**) Average time course of the excitatory effect in E13.5 MNs (n = 9). This excitatory effect was quantified every 20 ms by calculating the *R*_*GABA*_ as indicated in Suppl. Fig. 3. (**D**) Average time course of the inhibitory effect in E17.5 MNs quantified by *R*_*GABA*_ as in (C) (n = 10). The vertical bars represent the standard error of the mean (SEM). The significant differences of the R_GABA_ values compared to R_GABA_ at t = 0 ms are indicated by stars (*p < 0.05; **p < 0.01); nS: not significant. (**E,F**) IETC maps representing the time course (abscissa) of the threshold current (duration: 10 ms) during the simulation of a dGPSP in E13.5 (**E**) and E17.5 (**F**) neuron models as measured for a series of *E*_*Rest*_ (ordinate). Horizontal dashed lines represent the average of physiological *E*_*Rest*_. Vertical black dashed lines represent the simulated dGPSP peak. The magnitude of the excitatory (red colors) and inhibitory (cyan colors) effects is indicated by the color scale (% of change of the threshold current, see bottom bars for values). Contours are disposed at every 1% change. Identical rules hold true in all of the subsequent IETC maps. The time course of the simulated dGPSP is presented below each IETC map (same time scale). Green and red dashed vertical lines indicate the maximum excitatory and inhibitory effects, respectively. As in the following figures, the shape and size of the simulated neurons are presented in the insets on the IETC maps (proximal portion of the axon in blue).

We used whole-cell patch-clamp recordings while GABA_A_R responses were evoked by isoguvacine puff application at the soma level in E13.5 (Fig. 2A) and E17.5 MNs (Fig. 2B) that were loaded with E13.5 and E17.5 intracellular medium, respectively. These intracellular media were designed to fit the physiological E_Cl_ as measured in a gramicidin-perforated patch-clamp condition at similar developmental stages^17^. Although GABA_A_R and glyR are described as HCO3^−^ permeable^15^, we considered E_Cl_ as representative of E_GABAAR_/E_GlyR_ because of the low HCO_3_^−^ conductance of GABA_A_R in embryonic spinal MNs^22^. For these experiments, E_Rest_ was slightly more depolarized at E13.5 (−73.7 ± 1.9 mV, n = 6) than that at E17.5 (−85.8 ± 0.8 mV, n = 7). In contrast, E_Cl_ was dramatically more elevated at E13.5 (−44.0 ± 1.9 mV, n = 6) than that at E17.5 (−82.0 ± 2.3 mV, n = 5), thus revealing the higher [Cl^−^]_i_ in the E13.5 MNs compared with that of the E17.5 MNs (Table 1). Interestingly, at both developmental stages, the [Cl^−^]_i_ values were lower than the intra-pipette [Cl^−^], which was likely because of the high KCC2 efficacy in spinal MNs^17^. Given the difference in R_in_, our simulation study predicted that g_Clp_ must be increased 5.5-fold to obtain a comparable depolarization for similar E_Cl_ and E_Rest_ in both neurons (Suppl. Fig. 1A). In the experiments, isoguvacine puffs were adjusted to elicit a g_Clp_ of 1.6 ± 0.2 nS (n = 6) at E13.5 and a g_Clp_ of 5.2. ± 1.4 nS (n = 4) at E17.5 (Table 1) (see Supplemental Experimental Procedures). Note that we did not analyze the dGPSP falling phase because it outlasted the charge dissipation time course, indicating that it was a mixture of prolonged GABA_A_R activation by isoguvacine wave and charge dissipation (see Methods and Suppl. Fig. 2).

To evaluate the effect of dGPSPs, we developed a method based on the capacity of dGPSPs to modify the probability of threshold current pulses to evoke a spike. The intensity of the current pulses was adjusted to evoke a spike in approximately 50% of the trials prior to the evoked dGPSP. In this condition, an index termed R_GABA_ corresponding to the relative change in the spiking probability was calculated. This parameter represents the percentage of spikes that were triggered by depolarizing current pulses along the isoguvacine puff divided by the percentage of spikes that were triggered before the puff (see the Methods and Suppl. Fig. 3). At E13.5, prior to the isoguvacine puff, a spike was evoked in approximately 50% of the trials (Fig. 2A, left), resulting in R_GABA_ ≈ 1. The spikes were more steadily evoked along the rising phase of the dGPSP (Fig. 2A, right). Consequently, R_GABA_ increased from 0.90 ± 0.19 at t = 0 ms (response onset) to 1.53 ± 0.15 at t = 20 ms and then to 2.14 ± 0.17 at t = 60 ms (Fig. 2C). This finding indicates that the early depolarizing response to isoguvacine was exclusively excitatory (p < 0.001, n = 9, one-way ANOVA followed by Tukey’s multiple comparison test; see stars in Fig. 2C).

In contrast, at E17.5, using the same procedure, a purely inhibitory effect was observed during the rising phase of the dGPSP (Fig. 2B). R_GABA_ was 1.27 ± 0.09 at t = 0 ms (response onset) and significantly decreased (p < 0.05, n = 10, one-way ANOVA followed by Tukey’s multiple comparison test; see stars in Fig. 2D) to 0.28 ± 0.16 at t = 40 ms and 0.20 ± 0.13 at t = 80 ms (Fig. 2D). Thus, the rising phase of dGPSPs was purely inhibitory at E17.5.

We used computer simulations to analyze the complete dGPSPs (rising and falling phases) for a large range of E_Rest_. These simulations were used to construct a representation of the relative inhibitory/excitatory effect of dGPSP elicited on soma over time (inhibitory excitatory time course (IETC) maps, see Methods). The parameters were adjusted to fit the physiological experiments. The g_Clp_ values that were used in the E13.5- and E17.5-model neurons were 0.7 nS and 4 nS to respect the difference in input resistance (5.5-fold difference, see above). In the E13.5 neuron model, E_Cl_ was set to −42 mV. As shown in the IETC map (Fig. 2E), the dGPSP elicited a purely excitatory response at the physiological E_Rest_ (−74 mV, horizontal dotted line) and remained excitatory regardless of the E_Rest_ (from −85 mV to −65 mV). In contrast, simulations that were performed in the E17.5 neuron model with E_Cl_ set to −82 mV and g_Clp_ set to 4 nS (close to the g_Clp_ value that was used in the experiments) resulted in a purely inhibitory effect, regardless of E_Rest_ (Fig. 2F). Note that dGPSP was entirely inhibitory at the physiological E_Rest_ (−85 mV, horizontal dotted line) in the E17.5 neuron model.

It is generally admitted that the inhibitory effect of dGPSPs results from the shunting action carried by g_Cl_, whereas the excitatory effect is due to depolarization. Here, we sought to precisely assess the extent to which each component prevails during a dGPSP. We therefore quantified the excitatory and inhibitory dGPSP effects along the dGPSP time course. At E13.5, the excitation reaches it maximum at the dGPSP peak (Fig. 2E). In contrast, at E17.5, the inhibition was maximal 27 ms before the dGPSP peak (compare the red and green vertical dotted lines in Fig. 2F), which is consistent with the time course of g_Cl_ that carries inhibition (Suppl. Fig. 1B).

Thus, for E_Rest_ ranging from −85 to −65 mV, the GABA/glycine synapses were excitatory in the high-[Cl^−^]_i_ E13.5 neuron model and inhibitory in the low-[Cl^−^]_i_ E17.5 neuron model. Therefore, E_Cl_ appears to be determinant for the dGPSP effect. Interestingly, E_Cl_ is not a steady parameter, and during a high level of network activity, it tends to reach the membrane potential (Suppl. Fig. 4). In the following section, we analyze the effect of E_Cl_ on the dGPSP outcome. This physiological parameter was analysed in the two neurons that strikingly differ with respect to their passive features (*i.e*., size and R_in_).

### E_Cl_ plays a determinant role in the inhibitory/excitatory components of dGPSP

**Figure 3:**
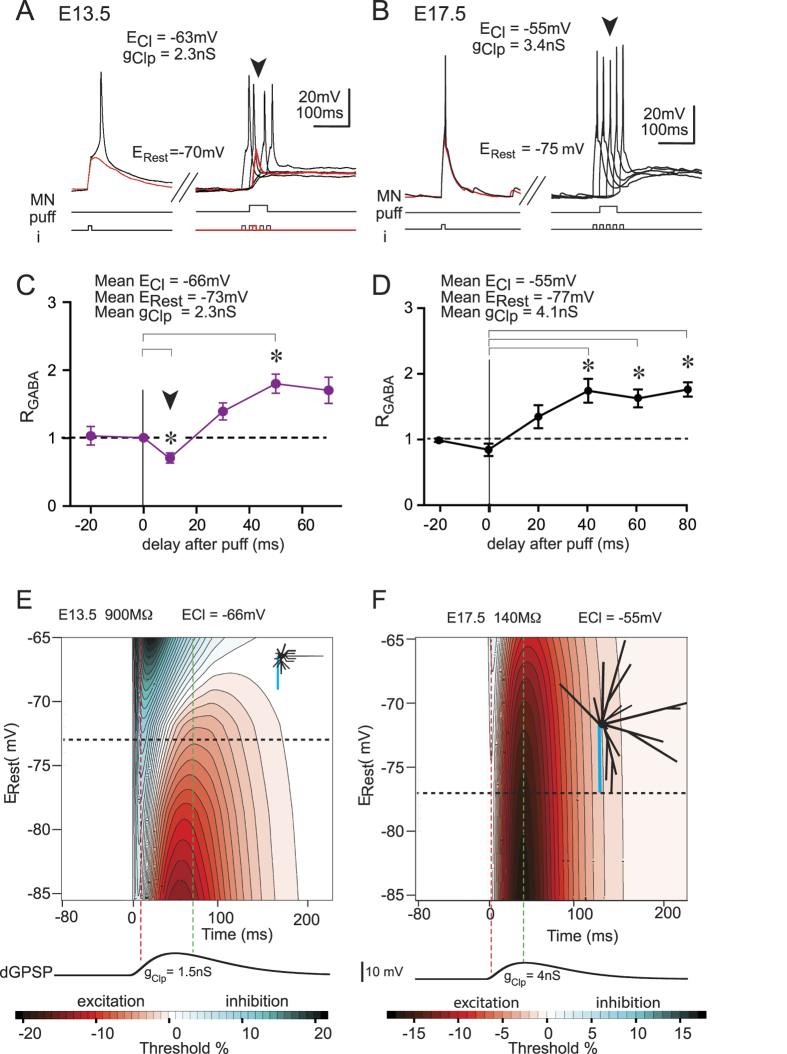
Time course of the excitatory and inhibitory effects of dGPSPs in E13.5 and E17.5 neurons with modified E_Cl_ values. (**A**,**B**) Patch-clamp recordings of an E13.5 MN (**A**) with *E*_*Cl*_ reduced to −63 mV and an E17.5 MN (**B**) with *E*_*Cl*_ increased to −55 mV during the injection of a threshold current pulse (i) in the absence (left traces) and presence (right traces) of an isoguvacine-induced response (puff). The arrowhead indicates inhibition (spike failure in A and spike amplitude reduction in B) at t = 10 ms after the initiation of MN responses to the isoguvacine puff. (**C,D**) Average time courses of the effects that were produced by isoguvacine in the E13.5 MNs (**C**, n = 5) and the E17.5 MNs (**D**, n = 10). Panels (**A,C)** are also presented in Fig. 5C,E. (**E,F**) IETC maps obtained from simulations using the parameters of the E13.5 (**E**) and E17.5 (**F**) neuron models with *E*_*Cl*_ values of −66 mV and −55 mV, respectively. The red and green vertical dashed lines represent the maximum inhibitory and excitatory effects, respectively, that were observed at the average resting membrane potential (horizontal black dashed line).

To verify whether E_Cl_ was able to alter the nature of dGPSPs independently of neuron morphology, E_Cl_ was reduced in the E13.5 MNs and increased in the E17.5 MNs. We found that the early excitatory action of dGPSP was abolished when E_Cl_ was reduced from −44.0 to −66 mV (−65.9 ± 2.1 mV, n = 4) in the E13.5 MN (Fig. 3A,C) (see Table 1 for the corresponding [Cl^−^]_i_). The g_Clp_ value (2.3 ± 0.1 nS, n = 4) was maintained within the same range as in Fig. 2C. Additionally, the mean value of E_Rest_ remained unaltered (−73.2 ± 3.8 mV, n = 4). Although the excitatory nature of the dGPSP remained (p < 0.01; one-way ANOVA for the dGPSP global time course), the R_GABA_ value showed a significant decrease at t = 10 ms (0.71 ± 0.07; p < 0.05; Tukey’s multiple comparison test; Fig. 3C), indicating an early inhibitory effect. Subsequently (t = 50 ms), the R_GABA_ value presented a significant increase compared with that of the control (1.80 ± 0.14, p < 0.05; Tukey’s multiple comparison test; Fig. 3C).

In contrast, the inhibitory GABA_A_R response that was observed at E17.5 with E_Cl_ = −82 mV was reversed to excitatory when E_Cl_ was increased to −55 mV (−55.3 ± 3.8, n = 5) (see [Cl^−^]_i_ in Table 1) (Fig. 3B,D) (p < 0.01; one-way ANOVA for the dGPSP global time course). R_GABA_ significantly increased at t = 40, 60 and 80 ms (p < 0.05; Tukey’s multiple comparison test; Fig. 3D). The g_Clp_ value (4.1 ± 0.8 nS, n = 4) was maintained in the same range as in Fig. 2D and E_Rest_ was −77.1 ± 1.7 mV (n = 6).

These results indicate that both neurons exhibit similar dGPSP effects when they share comparable E_Cl_ and E_Rest_ values. To extend our analysis to a large range of E_Rest_ values, simulations based on the above physiological parameters were conducted (Fig. 3E,F). The E13.5 neuron model exhibited a slight inhibition at the onset of the dGPSP (see the blue region on the IETC map) when its E_Rest_ was set to the physiological E_Rest_ = −73 mV (horizontal dotted line in Fig. 3E), with E_Cl_ = −66 mV and g_Clp_ = 1.5 nS. At this E_Rest_ potential, the magnitude of inhibition and excitation was weak (9% and 6%, respectively); however, their duration strongly differed (33 and 130 ms, respectively) (Fig. 3E). These results are consistent with the corresponding physiological data. Note that the early inhibition that was observed as a reduction in the spike amplitude (see arrowhead in Fig. 3A,B) is now explained by the presence of the short inhibition that was present at the onset of the dGPSPs. This inhibition delays the R_GABA_ increase (compare Fig. 3C with Fig. 2C for the 20-ms pulse delay).

In conclusion, we demonstrate that E_Cl_ plays a crucial role in the control of the inhibitory *versus* the excitatory nature of dGPSPs. Therefore, a pure excitatory dGPSP becomes a mixed inhibitory/excitatory dGPSP when the E_Cl_ decreases from −42 mV (Fig. 2E) to −66 mV (Fig. 3E). Similarly, a pure inhibitory dGPSP becomes a mixed inhibitory/excitatory dGPSP when the E_Cl_ increases from −82 mV (Fig. 2F) to −55 mV (Fig. 3F). Although the [Cl^−^]_i_ was altered in both of the model neurons to achieve the same E_Cl_ value, experimental measurements revealed a difference (−66 mV for E13.5 and −55 mV for E17.5). This difference may be responsible for the larger excitation and lower inhibition that were observed in the E17.5 model neuron compared with the E13.5 model neuron; however, it may also be related to neuron passive properties that differ between both neurons. To determine the role of neuron passive properties in the dGPSP effects, new simulations with identical E_Cl_ values were conducted.

### Role of neuron features in the inhibitory/excitatory components of dGPSPs

**Figure 4:**
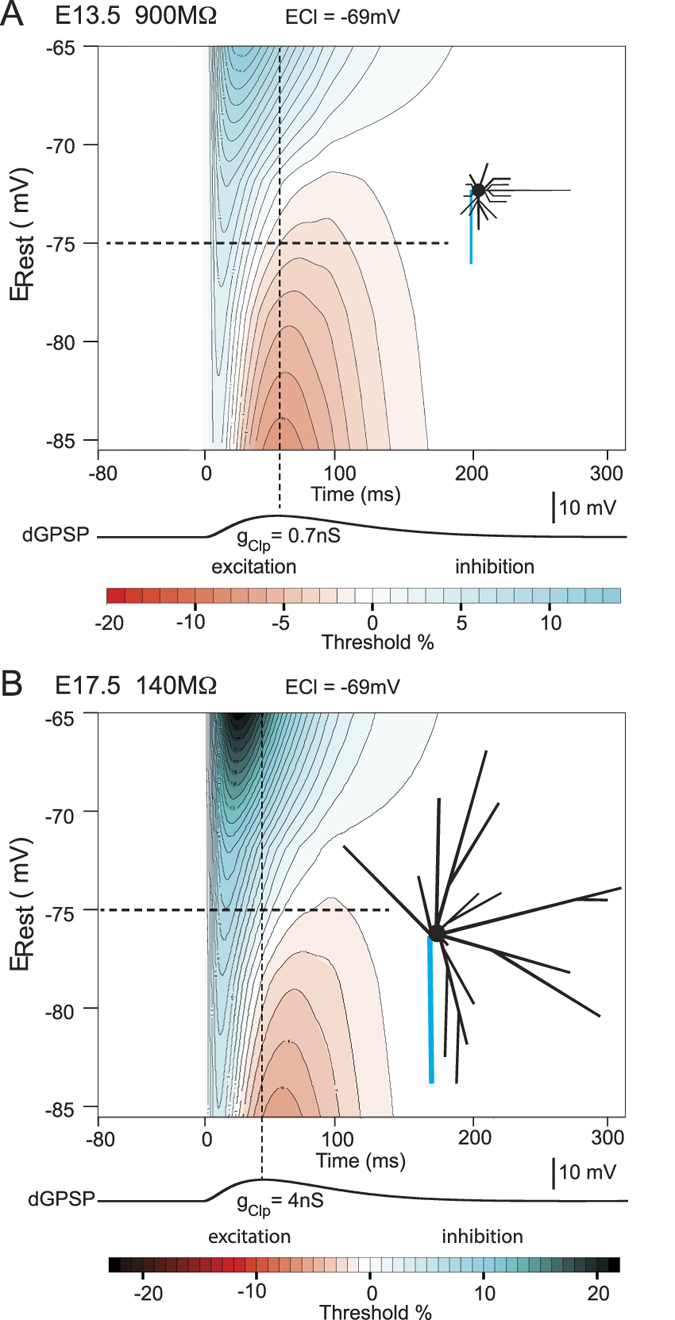
IETC maps generated from E13.5 (A) and E17.5 (B) neuron models with similar E_Cl_ values (−69 mV). The conductance (*g*_*Clp*_) of the simulated GABA_A_R synapse was adjusted to elicit the same depolarization (7 mV) in both of the neuron models. The vertical black dashed lines indicate the peak in GPSP depolarization. The horizontal black dashed lines represent the average resting potential.

The functional consequences of neuron passive properties (*i.e*., R_in_ and size) on the inhibitory/excitatory dGPSP components were studied by setting E_Cl_ to −69 mV for both of the model neurons (Fig. 4). A similar dGPSP amplitude at E_Rest_ = −75 mV was obtained using a g_Clp_ of 0.7 nS for the E13.5 neuron model and of 4 nS for the E17.5 neuron model (Suppl. Fig. 1). The results indicate that inhibition always exists at the onset of the dGPSP regardless of E_Rest_ (from −85 mV to −65 mV). For E_Rest_ < −75 mV, the inhibition was followed by an excitatory component. However, the comparison of IETC maps for the E13.5 and E17.5 neuron models revealed three slight differences due to neuron features. First, when E_Rest_ < −75 mV, the duration of excitation is greater at E13.5 (Fig. 4A) than that at E17.5 (Fig. 4B). Second, the amount of inhibition is smaller at E13.5 (the threshold current increased by +14%) than that at E17.5 (the threshold current increased by +23%) (see the blue region in Fig. 4A,B). Third, although at E17.5, no excitation was detected for E_Rest_ > −75 mV, the excitation at E13.5 remains a few millivolts above E_Rest_ = −75 mV (see the upper limit of the excitatory domain in Fig. 4A). These results indicate that in large neurons with low resistance, inhibitory dGPSP effects are moderately favored. However, the effect of passive properties remains minor compared with the effect of E_Cl_.

The substantial similarities in the IETC maps indicate that our analysis can be extended to a wide diversity of neuronal types (see Fig. 1). Subsequent simulations were performed on the E13.5 neuron model that, in spite of a weakened inhibitory shunting effect, demonstrates a full capacity to switch from excitation to inhibition (see below). The next parameter studied was g_Clp_. Up to this point, this parameter was set according to neuron passive properties to adjust the dGPSP amplitude (Suppl. Fig. 1). We then evaluated the functional consequences of modifying g_Clp_ in the E13.5 neuron model.

### Increasing g_Clp_ switches excitatory dGPSPs to inhibitory dGPSPs

**Figure 5:**
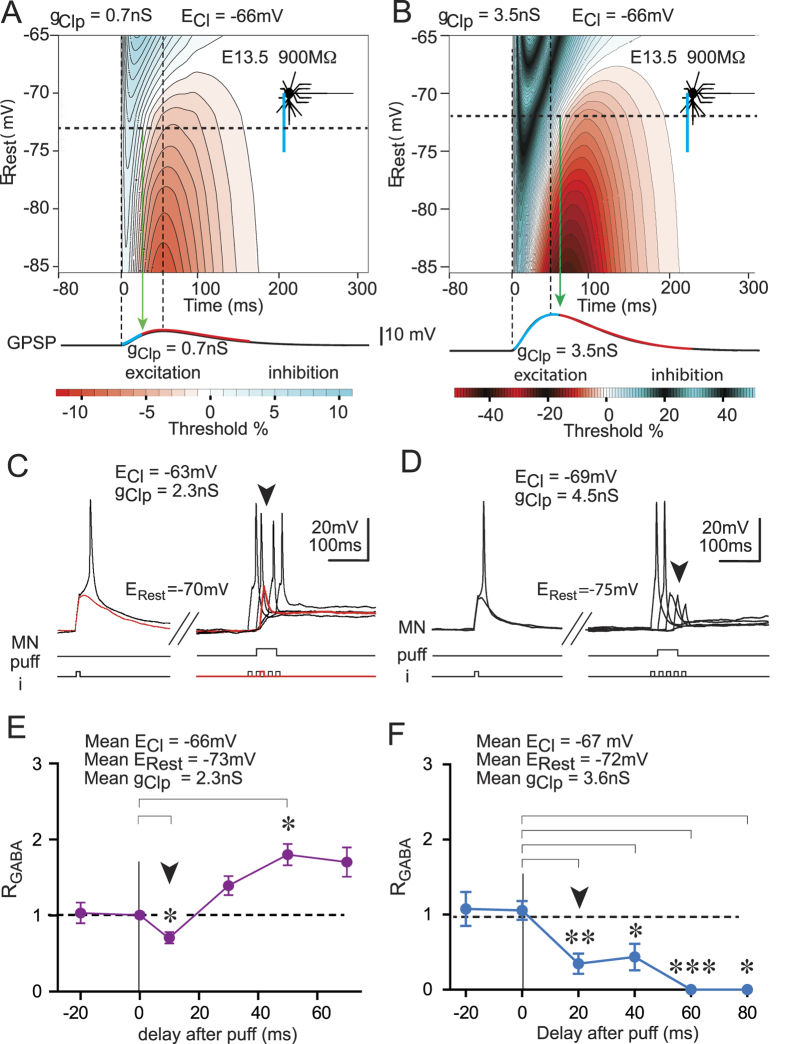
Effect of increasing the g_Clp_ in the E13.5 neuron model is verified in physiological experiments. (**A,B**) IETC maps generated from the E13.5 neuron model for a *g*_*Clp*_ of 0.7 nS (**A**) and 3.5 nS (**B**). The limit between the inhibitory effect (in blue on the dGPSP trace) and the excitatory effect (in red on the dGPSP trace) for a resting membrane potential of −73 mV (horizontal dashed line) is indicated by a green vertical arrow. This limit increases from 50% to 100% of the dGPSP rising phase when the *g*_*Clp*_ is increased from 0.7 nS to 3.5 nS. The vertical dashed lines represent the onset and peak of dGPSP. (**C,D**) Effect of increasing isoguvacine release on the E13.5 MNs. A biphasic early inhibitory (at t = 10 ms) and late excitatory effect is elicited with a low GABA_A_R conductance activation (*g*_*Clp*_ = 2.3 nS, **C**). Note that the excitatory effect is much larger than the inhibitory effect. However, a purely inhibitory effect occurs with a larger conductance (*g*_*Clp*_ = 4.5 nS, **D**). (**E,F**) Average excitatory and inhibitory responses that were produced by a low conductance (mean *g*_*Clp*_ = 1.8 nS, **E**) and a large conductance (mean *g*_*Clp*_ = 3.5 nS, **F**). Panels (**C,E**) are the same as in Figs 3A and 5C. The responses were quantified by calculating the *R*_*GABA*_ values (n = 5 in **E** and n = 5 in **F**). Significant differences compared to the control *R*_*GABA*_ (t = 0) are indicated by stars (*p < 0.05; **p < 0.01; ***p < 0.001; nS: not significant).

Increasing g_Clp_ increases both the amplitude of the dGPSP depolarization (excitatory effect) and the shunting (inhibitory effect). Which effect would then dominate, excitation or inhibition? To address this question, we evaluated the effect of increasing g_Clp_ in simulations. E_Cl_ was set at −66 mV to generate IETC maps with balanced excitatory and inhibitory effects. We found that increasing g_Clp_ from 0.7 nS to 3.5 nS increased the amplitude and duration of both the inhibitory and the excitatory components of dGPSPs (compare Fig. 5A,B). However, increasing g_Clp_ differentially affected the intensity of the two components: for example, at E_Rest_ = −72 mV (horizontal dotted line), the excitation increased from 4% to 5%, whereas the inhibition increased from 3% to 26%, favoring inhibition rather than excitation. The duration of inhibition also increased when g_Clp_ was increased. For example, at E_Rest_ = −73 mV, 50% of the dGPSP rising phase was inhibitory for g_Clp_ = 0.7 nS and 100% for g_Clp_ = 3.5 nS (compare the blue region of the dGPSP time course in Fig. 5A,B).

**Figure 6:**
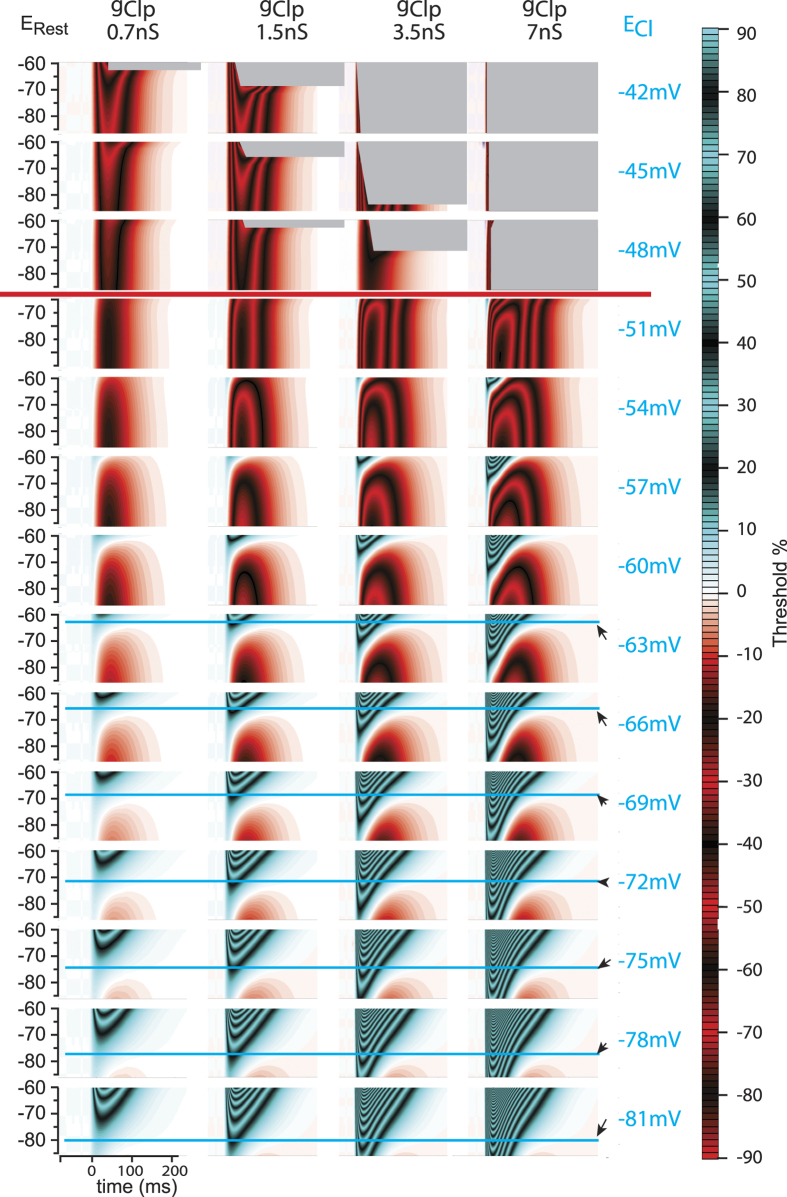
IETC maps for E_Cl_ ranging from −81 mV to −42 mV and four g_Clp_. The red horizontal line indicates the separation between the suprathreshold and subthreshold values of E_Cl_. On each IETC map, the blue horizontal line indicates when the membrane potential reaches the E_Cl_ value for that IETC map. The gray regions represent the domains in which spikes are elicited by suprathreshold dGPSPs.

We then evaluated whether these predictions were observed in physiological experiments. Depolarizing GPSPs were produced in E13.5 MNs with E_Cl_ reduced approximately to −66 mV (−67.8 ± 1.0, n = 3) (as in Fig. 3A) (see [Cl^−^]_i_ in Table 1), and g_Clp_ increased from 2.3 nS (Fig. 5C,E) to 3.6 nS (3.6 ± 0.4, n = 3) (Fig. 5D,F). Interestingly, this increase in g_Clp_ resulted in a significant inhibition (p < 0.0001; one-way ANOVA) during the rising phase of the dGPSP: R_GABA_ significantly decreased from 1.06 ± 0.12 prior to the dGPSP to 0.35 ± 0.13 at t = 20 ms and to 0.00 ± 0.00 for t ≥60 ms (Tukey’s multiple comparison test) (see the stars in Fig. 5F). Therefore, increasing g_Clp_ results in a switch from excitation to inhibition for an identical E_Cl_ value. This result validates the prediction from computer simulations that were performed using E_Cl_ = −66 mV and E_Rest_ = −73 mV (−72.6 ± 1.2, n = 3 in real MNs) (Fig. 5A,B). This prediction holds true for E_Cl_ values ranging from −81 mV to −60 mV (Fig. 6).

### Trains of spike-triggering dGPSPs switch from excitation to inhibition with increasing frequency

Until now, we have considered E_Cl_ values that did not allow dGPSPs to elicit spikes (in the absence of depolarizing current pulses). For more depolarized E_Cl_ values, dGPSPs may trigger spikes (see IETC maps for E_Cl_ > −51 mV in Fig. 6). In this case, it remains uncertain whether increasing g_Clp_ could still enhance inhibition because of spike(s) generation. If this is the case, both excitatory and inhibitory effects should coexist and overlap during dGPSP trains. To evaluate this hypothesis, we designed simulations in which over-threshold dGPSPs overlap during trains. The functional consequence of this overlap was then evaluated. Would a train result in excitation or inhibition?

**Figure 7:**
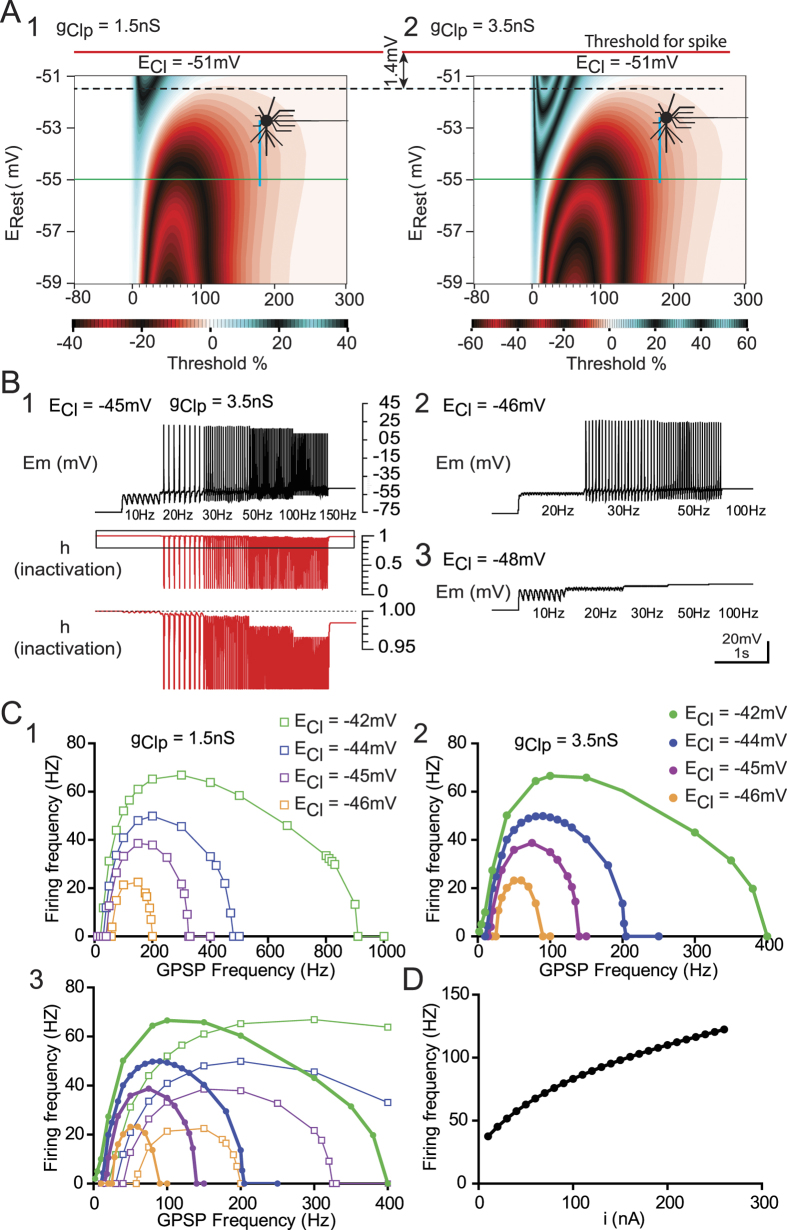
The switch from excitatory to inhibitory dGPSP barrage is frequency dependent. (**A)** IETC maps were generated for E_Cl_ (−51 mV) close to the spike threshold E_Thr_ (−50 mV, red horizontal line) and for resting membrane potentials in the vicinity of the spike threshold. The early inhibitory effect of dGPSPs increases more rapidly in the vicinity of the spike threshold for a large *g*_*Clp*_ (3.5 nS, **A**_**1**_) than for a small *g*_*Clp*_ (1.5 nS, **A**_**2**_), whereas the excitatory effect virtually does not change. (**B)** Effect of the dGPSP train rate on the evoked discharge for three *E*_*Cl*_ values: with *E*_*Cl*_ = −45 mV (**B**_**1**_) spikes are elicited when the dGPSP frequency reaches 20 Hz, and the discharge is blocked at a dGPSP train rate >140 Hz; with a more hyperpolarized *E*_*Cl*_ value (−46 mV, **B**_**2**_), the range for spike triggering is reduced (between 30 and 50 Hz); for an *E*_*Cl*_ value of **−**48 mV (**B**_**3**_), no spikes are elicited regardless of the dGPSP train rate. Note that the suppression of the spike discharge at a high dGPSP rate is not due to the inactivation (*h*) of voltage-dependent Na^+^ channels that remains very close to 1 (see the red traces in **B**_**1**_) even at the highest dGPSP frequency (150 Hz). (**C)** Firing rate as a function of dGPSP frequency for *g*_*Clp*_ = 1.5 nS (**C**_**1**_) and *g*_*Clp*_ = 3.5 nS (**C**_**2**_). The two curves are superimposed in (**C**_**3**_). (**D)** In contrast to the bell-shaped curves of the firing rate *versus* the dGPSP frequency, the firing frequency response to current injection is a monotonic curve.

The first step was to confirm whether inhibition was present in a single dGPSP occurring in the vicinity of E_Thr_. In this vicinity, the trains of subthreshold dGPSPs leading to summation eventually reach E_Thr_. What would the effect be of increasing g_Clp_ in this voltage range? Additionally, over-threshold dGPSPs with high g_Clp_ values produce spikes at a lower E_Rest_ (Fig. 6). Increasing g_Clp_ should therefore favor excitation when approaching E_Thr_. However, we have demonstrated that increasing g_Clp_ in dGPSPs favors inhibition. To resolve this contradiction, single dGPSPs were simulated with E_Rest_ between −59 mV and −51 mV (Fig. 7A,B). E_Cl_ was set to −51 mV to prevent the generation of a spontaneous spike by a dGPSP. The IETC map, which was generated with g_Clp_ = 1.5 nS, revealed the existence of an early inhibitory domain that rapidly increased from 5% to 27% when E_rest_ was depolarized from −59 mV to inhibitory domain that rapidly 51 mV (Fig. 7A1). When g_Clp_ was increased from 1.5 nS to 3.5 nS (Fig. 7A_2_), the early inhibitory dGPSP component reached a maximum of 60%, whereas the excitatory component was less affected and increased from 37% to 44%. This result indicates that rising g_Clp_ still favors inhibition when E_Rest_ approaches E_Thr_.

As a second step, for more depolarized E_Cl_ values, we evaluated whether dGPSP-induced spikes could be prevented by increasing g_Clp_. We performed a new series of simulations with E_Rest_ = −75 mV, in which dGPSPs reached E_Thr_ and elicited spikes. In these simulations, we explored the relationship between the dGPSP frequency and the firing response of the neuron model. As illustrated in Fig. 7B1, with E_Cl_ = −45 mV and g_Clp_ = 3.5 nS, a 10-Hz dGPSP train did not elicit action potential, with the E_m_ remaining < E_Thr_. When the dGPSP train frequency was increased to 20 Hz, spikes were elicited. This limit frequency was termed the “switch-on” frequency. When the dGPSP train frequency was further increased to 100 Hz (Fig. 7B1), the firing frequency accordingly increased up to ~35 Hz. Finally, increasing the dGPSP train frequency to 140 Hz (Fig. 7B1) completely inhibited the spike discharge. This dGPSP train frequency was termed the “switch-off” frequency. The “switch-off” frequency was not due to voltage-dependent Na^+^ channel inactivation because simulations were performed with HH parameters, limiting the inactivation of Na^+^ channels (see the simulation methods for Na^+^ channels). This limitation is shown in Fig. 7B1 (red traces), in which the inactivation h value is monitored. When the dGPSP rate reached 140 Hz, the h value was nearly unchanged (0.985 instead of 1, *i.e*., nearly no inactivation). Decreasing the E_Cl_ from −45 mV to −46 mV produced similar results; however, the dGPSP frequency window for spike production was narrowed (Fig. 7B2). A further reduction of the E_Cl_ to −48 mV resulted in the inability for dGPSPs to elicit spikes (Fig. 7B3). These simulations were repeated for five E_Cl_ values (−48 mV, −46 mV, −45 mV, −44 mV and −42 mV) and 2 g_Clp_ values (1.5 and 3.5 nS), and frequency response curves were generated (Fig. 7C). These simulations demonstrated that the frequency response curve was always bell-shaped, firing between “switch-on” and “switch-off” frequencies, with a maximum spike frequency increasing with more depolarized E_Cl_. Interestingly, simulations revealed that this maximum frequency was not g_Clp_-dependent (compare Fig. 7C_1_,C_2_). The bell-shaped response was narrower, and the “switch-on” frequency was lower for the high g_Clp_ value (3.5 nS) than for the low g_Clp_ value (1.5 nS) (Fig. 7C3).

We have demonstrated that the “switch-off” frequency is not due to voltage-dependent Na^+^ channel inactivation (see above). This frequency is also not due to the activation of K^+^ channels, which would limit the frequency discharge. Indeed, when a current was injected in the E13.5 neuron model, the discharge frequency monotonically increased with the current intensity (Fig. 7D). Therefore, the shape of the frequency response curve with a “switch-off” frequency is a property of dGPSPs and results from the increasing strength of the inhibitory component of dGPSPs.

**Figure 8:**
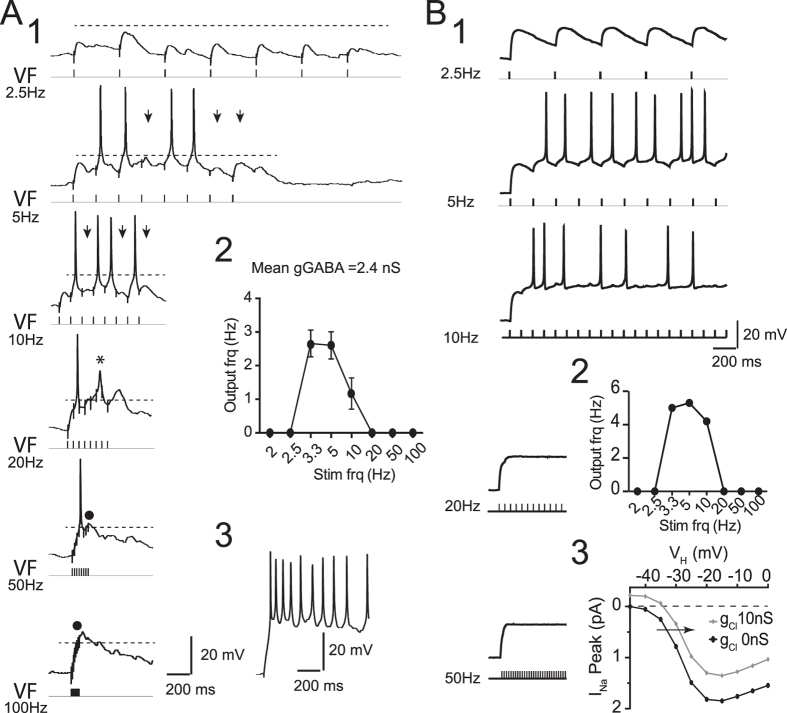
Experimental demonstration of the switch from excitatory to inhibitory dGPSP barrage with increasing frequency. (**A**_**1**_) Whole-cell patch-clamp recording from an E13.5 MN (E13.5 intracellular medium) while stimulating the ventral funiculus (VF) at various frequencies in the presence of AP5 (20 μM), CNQX (50 μM) and DHβE (5 μM) (E_Rest_ = **−**74 mV). Each VF stimulation produced a dGPSP (mean conductance of 2.4 nS). At 2.5 Hz, the dGPSP did not elicit a spike. At 5 Hz and 10 Hz, the dGPSP summation occurred and the E_Thr_ (horizontal dashed line) was reached. However, because of the amplitude variability among the dGPSPs, certain VF stimulations failed to reach E_Thr_ (arrows). At 20 Hz, an action potential was elicited by the second VF stimulation. The following stimuli (steady state) failed to elicit spikes despite reaching E_Thr_. Note that an aborted spike was observed (asterisk). At 50 Hz, only one spike was produced on the fourth stimulus, whereas at 100 Hz, the VF stimulation did not induce an action potential (black dot). (**A**_**2**_) Average I/O frequency relationship calculated from five E13.5 MNs. (**A**_**3**_) Spontaneous burst of activity demonstrating that the MN is able to fire at a frequency reaching 20 Hz. (**B**_**1**_) Simulation of the E13.5-model MN with the same characteristics as the real MN presented in A-B. VF stimulation was mimicked by a 2.5 nS synaptic conductance and ECl set to −43 mV with the same kinetic as the VF-evoked synaptic current (rising time constant 2 ms, decay time constant 150 ms). In this simulation, the inactivation of Na^+^ channels was <1.5% as in Fig. 7B. (**B**_**2**_) Bell-shaped I/O frequency relationship. (**B**_**3**_) Curves of peak sodium channel currents (I_Na_Peak) against the imposed membrane potential (V_H_, simulated voltage-clamp) in the absence (black) and the presence (grey) of a constant 10 nS g_Cl_.

We then designed an experimental procedure to validate this prediction. GABA/glycinergic synapses were activated by electrical stimulation of the ventral funiculus (VF)^23^ in E13.5 SC after blocking the glutamatergic and cholinergic synaptic components (see Supplemental Experimental Procedures). To avoid transient collapse of the chloride reversal potential (Suppl. Fig. 4), brief trains (8 pulses) were used. When stimulated at low frequency (2,5 Hz), the VF evoked the under-threshold dGPSPs (Fig. 8A1). At 5 Hz–10 Hz, temporal summations of the dGPSPs produced spikes in approximately 50% of stimuli. Higher frequency stimulations did not evoke spikes in the steady-state regime (Fig. 8A1). The average I/O frequency relationship, which was calculated from five E13.5 MNs, was bell shaped (Fig. 8A_2_). Up to 2.5 Hz, a spike was not produced; between 3.3 and 5 Hz, the output firing reached a plateau (2.6 ± 0.4 Hz, n = 29 from 5 experiments); at 10 Hz, the output frequency decreased to 1.2 ± 0.4 Hz (n = 22 from 5 experiments); and at 20 Hz and higher, a spike did not occur in the steady state. Note that the low output frequency (<3 Hz) was not caused by a limitation of the MN firing frequency because a spontaneous burst of activity could reach up to 20 Hz (Fig. 8A3). To validate the hypothesis that the increasing inhibitory component was responsible for the switch from excitation to inhibition when the VF frequency was increased, we performed a series of simulations using the E13.5-model MN (Fig. 8B) with the same characteristics as the real MNs presented in Fig. 8A1-2 (see measured physiological parameters in Table 2). VF was mimicked by a 2.5 nS synaptic conductance on soma and E_Cl_ set to −43 mV with the same kinetics as a VF-evoked synaptic current (rising time constant 2 ms, decay time constant 150 ms). Note that this time constant was much longer than the one used to mimic the single sGPSP (20 ms, see Supplemental Experimental Procedures). As expected, increasing the input dGPSP frequency led to an initial excitatory effect (because of dGPSP summation) followed by a complete inhibition of the output discharge at the VF-like stimulation frequency ≥20 Hz (Fig. 8B1-2). The inhibitory effect of high-rate GPSP trains resulted from the shunt exerted by the GPSP summation that shifted the curve of the peak sodium channel current (I_Na_Peak) towards more positive membrane potential values (see horizontal arrow in Fig. 8B3). The shifted curve (grey) illustrates the effect of a 10 Hz dGPSP train leading to a shunt summation of ~10 nS (see eq. (1)). Thus, for the same membrane potential, the amount of activated Na^+^ channels was reduced by the shunting effect of the dGPSPs, leading to spike failure.

### Functional consequences of dGPSP trains on excitatory drive

**Figure 9:**
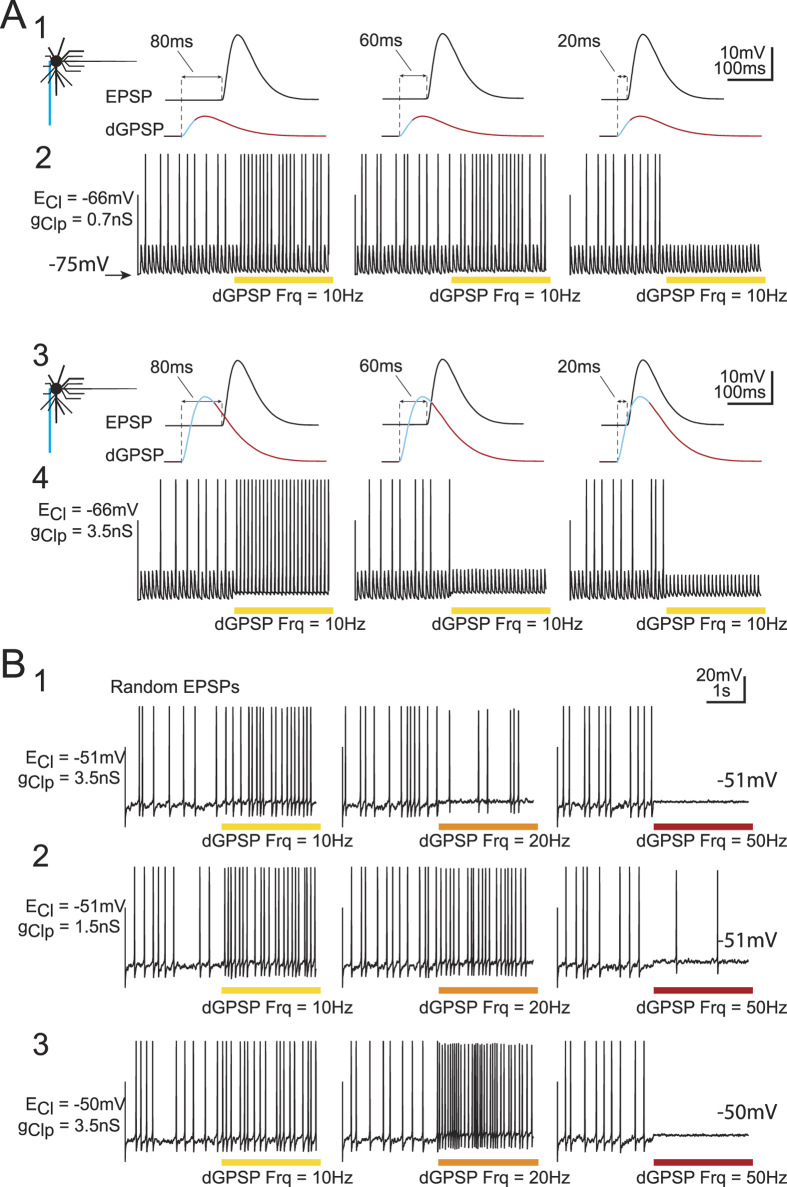
Interaction between dGPSP trains and excitatory drive. (**A**) Phase-locked dGPSPs/EPSPs at three fixed delays (see top insets) at 10 Hz for *g*_*Clp*_ = 0.7 nS (**A**_**1**_**,A**_**2**_) and *g*_*Clp*_ = 3.5 nS (**A**_**3**_**,A**_**4**_). E_Cl_ is set to −66 mV in (**A**). Synaptic noise was added to the model to allow some subthreshold EPSPs to evoke spikes. For large delays (*i.e*., when EPSP occurs late in the repolarizing phase of the dGPSP), the spike probability is increased, whereas for small delays, an inhibitory effect is observed. The transition from excitation to inhibition is more abrupt when *g*_*Clp*_ is increased from 0.7 nS (**A**_**1**_**,A**_**2**_) to 3.5 nS (**A**_**3**_**,A**_**4**_). (**B**) Unsynchronized high rate EPSP barrage for three dGPSP frequencies (10, 20 and 50 Hz) and three *E*_*Cl*_/*g*_*Clp*_ conditions. Random EPSP barrage results in a random spike triggering. When the dGPSP train is switched on at 10 Hz, the spike probability increases (excitatory effect), whereas the 50-Hz dGPSP train is inhibitory. The inhibitory effect is stronger for the large *g*_*Clp*_ value (3.5 nS, **B**_**1**_) than for the small *g*_*Clp*_ value (1.5 nS, **B**_**2**_), whereas the switch from an excitatory to inhibitory effect occurs more abruptly for the more depolarized *E*_*Cl*_ value (*E*_*Cl*_ = **−**50 mV, **B**_**3**_).

Spinal motor neurons generally receive concomitant excitatory and inhibitory drives; however, during such concomitant drives, excitatory and inhibitory PSPs are generally not synchronized, although in other networks, such as cortical networks, EPSP/IPSP synchronization is a rule^24^. To explore how both drives interact, we performed new simulations in which the same neuron model at E_Rest_ = −75 mV received concomitant trains of purely excitatory glutamate-like EPSPs and dGPSPs (Fig. 9). A preliminary series of data illustrates the effects of EPSPs occurring at fixed delays relative to dGPSPs (80 ms, 60 ms, and 20 ms; see schemas in Fig. 9A1). In these simulations, E_Cl_ was set to −66 mV, a value for which inhibitory and excitatory domains coexist at E_Rest_ of approximately −75 mV (Fig. 5A,B). Large EPSPs were generated at a rate of 10 Hz. The amplitude of EPSPs fluctuated slightly using a random generator (see Methods) such that the 10-Hz EPSP train could trigger spikes with an average rate of 4 Hz (Fig. 9A_2_,A_4_). After 2.5 s of the EPSP barrage, a dGPSP train was added at the same rate as that of the EPSP train but with a constant delay. Two g_Clp_ values were evaluated: a low (0.7 nS, Fig. 9A_2_) and a high (3.5 nS, Fig. 9A3,A4) value. dGPSPs occurring with long delays prior to EPSPs were excitatory. This effect switched to inhibition for shorter delays in a g_Clp_-dependent manner, consistent with the corresponding IETC maps (Fig. 5), which quantify the magnitude and duration of the inhibitory *versus* excitatory components of dGPSPs. These two components are indicated on the dGPSP traces, as shown in Fig. 9A1,A3.

In spinal motor networks, concomitant excitatory and inhibitory drives are generally not synchronized at the level of PSPs. Therefore, in a final series of simulations that were performed in the same neuron model, EPSPs were randomly elicited according to Destexhe *et al*.^25^, in which EPSP conductance (0.4 ± 0.1 nS) resulted in a mean E_m_ of approximately −55 mV. At this membrane potential, a few spikes were sparsely triggered (4 Hz, Fig. 9B1). To elicit dGPSPs, E_Cl_ was set to −51 mV (subthreshold). Again, low (1.5 nS) and high (3.5 nS) g_Clp_ values were used. With the high g_Clp_ value, a 10-Hz dGPSP train resulted in a slight excitatory effect (the spike frequency increased from 4 Hz to 7.2 Hz, Fig. 9B1, left). When the dGPSP rate increased to 20 Hz, the spike frequency decreased to 2.4 Hz (Fig. 9B1, middle). A further dGPSPs rate increase to 50 Hz led to a complete inhibition of the discharge (Fig. 9B1, right). The switch from an excitatory to inhibitory effect is consistent with IETC maps in the vicinity of E_Thr_ (Fig. 7A). The average effect of a dGPSP train is dependent on the relative duration of the inhibitory and excitatory domains. As illustrated in Fig. 7A1, for E_Cl_ = −51 mV and E_Rest_ = −55 mV (green line), the early inhibitory domain of each dGPSP is transient and lasts <25 ms. This inhibitory effect is then followed by an excitatory effect lasting ∼250 ms. dGPSP train frequencies of greater than 50 Hz (*i.e*., 20 ms between the onset of successive dGPSPs) resulted in inhibition due to the overlap of dGPSP inhibitory domains. Moreover, conductances of successive dGPSPs summate following the law of exponential decay summation (see Methods). This law is formalized by the following equation (1) giving the limit of summated conductances (g_Peaklim_):


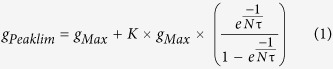


in which *N* is the dGPSP train frequency, *τ* is the time constant of dGPSP decay, and *K* is the coefficient that is used to assimilate the dGPSP to a single exponential decay curve (beginning from a theoretical initial value of 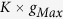, Suppl. Fig. 5) see^26^ for a demonstration. With N = 50 Hz, *τ* = 20 ms, K = 2.4 and g_Max_ =  = 3.5 nS, g_Peaklim_ is dramatically increased to 8.39 nS. In contrast, the depolarization of dGPSPs is limited by an E_m_ value that is close to E_Cl_ (set to −51 mV), whereas shunting continues to summate and results in a net inhibitory effect (see Fig. 9B1, right). A decrease in g_Clp_ from 3.5 nS to 1.5 nS resulted in an excitatory effect of dGPSP trains at 10 Hz and 20 Hz (from 4 to 8.2 Hz and 8.4 Hz, respectively, Fig. 9B2, left and middle), which is consistent with the shortening and intensity reduction (17% to 6%) of the transient inhibition occurring at dGPSP onset (Fig. 7A). Moreover, because the maximum excitation only decreased from 25% to 22% (Fig. 7A) when g_Clp_ decreased from 3.5 nS to 1.5 nS, the balance between inhibition and excitation favors the excitatory effects of 20-Hz dGPSP trains, as illustrated in Fig. 9B2 (middle). At 50 Hz, dGPSP trains do not completely prevent firing (Fig. 9B2, right) because g_Peaklim_ reaches a lower value (3.59 nS instead 8.39 nS for g_Clp_ = 3.5 nS).

In conclusion, g_Clp_ is the key parameter that controls the switch from excitatory to inhibitory dGPSP trains for subthreshold E_Cl_. However, does this action persist if E_Cl_ remains subthreshold but is more depolarized? To address this question, E_Cl_ was slightly depolarized to −50 mV (close to E_Thr_) with g_Clp_ = 3.5 nS. Figure 9B3 shows that dGPSP trains remain excitatory at 10 Hz (the spike frequency increased from 4 to 8.2 Hz, Fig. 9B3, left). This excitatory effect was dramatically enhanced at 20 Hz (the spike frequency reached 14.8 Hz, Fig. 9B3, middle). At 50 Hz, the dGPSP train effect switched to complete inhibition (Fig. 9B3, right). Thus, when the subthreshold E_Cl_ approaches E_Thr_, the dGPSP effects on the excitatory drive become more contrasted, switching from a large excitation at a low frequency (20 Hz) to an abrupt inhibition at a higher frequency (50 Hz).

## Discussion

By studying the interaction between the excitatory and inhibitory effects of depolarizing GABA/glycine synaptic events, we have demonstrated that 1) single dGPSPs express an inhibitory component that increases with membrane depolarization; 2) this increase is potentiated by large chloride conductance; 3) even when E_Cl_ approaches the spiking threshold, the inhibitory component remains present and interestingly prevails when the membrane potential approaches the firing threshold; 4) when E_Cl_ crosses the spiking threshold, although the inhibitory component does not prevent spikes for small chloride conductance values, it prevents spiking activity for larger chloride conductance values (Fig. 7A); and 5) during trains of spike-inducing dGPSPs, the inhibitory component of dGPSPs is potentiated with a high frequency and eventually prevents spiking discharge (Fig. 7B,C). Therefore, the excitation/inhibition duality in dGPSPs is dependent not only on E_Cl_, g_Clp_, and E_Rest_ but also on the discharge frequency of dGPSPs.

From a functional perspective, when dGPSPs interact with EPSPs, two domains must be considered: 1) in a low-frequency domain in which dGPSPs and EPSPs are time-locked, the delay between both types of synaptic events controls the switch from excitation to inhibition, and 2) in a high frequency domain, dGPSPs are no longer correlated with EPSPs, and the switch from excitation to inhibition is only dependent on the dGPSP frequency. Interestingly, this pattern holds true even when the E_Cl_ is above the threshold.

### Spatial localization of dGPSP effects

Our study concerned the local (somatic) effect of dGPSPs. However, dGPSPs may exert differential spatial effects. This possibility has been demonstrated in postnatal rat spinal MNs in which a depolarizing GABA/glycine-mediated PSP exerts an excitatory or inhibitory effect depending on its location and E_Cl_ value^13^. The inhibitory effect of the dGPSPs that are located on dendrites decreases with the distance to the soma and may represent a refined process that regulates the integrative properties of the neuron. Following spinal cord injuries, E_Cl_ undergoes a shift toward more depolarized values that enhance excitatory effects of dGPSPs and may be responsible for spasticity^27^. A similar spatial mechanism appears to be involved in cortical layer 5 pyramidal neurons during “up” states when the membrane potential is just below E_Cl_: dGPSPs exert a local shunting inhibition on apical dendrites but promote a distant excitatory effect on basal dendrites^1^. Our demonstration that local dGPSPs switch from excitatory to inhibitory at a high frequency is true regardless of the dGPSP location. However, considering the distant effects of these dGPSPs, we must dissociate their inhibitory component that remains local from their excitatory component that spreads electrotonically over long distances.

### Functional relevance of dGPSPs during CNS development

Depolarizing GPSPs represent key elements in immature brain structures in which they play an important role in the construction of neural networks. In most immature neurons, E_Cl_ is highly depolarized in early development stages^17^,^28^,^29^,^30^,^31^,^32^,^33^,^34^,^35^,^36^,^37^. In E12.5 embryonic mouse spinal MNs, GABA exerts, with glutamate (Glu) and acetylcholine (Ach), excitatory effects that primarily support spontaneous activity waves^9^. These waves are required for correct motor axon guidance^38^. In rat embryonic hypothalamic neurons^14^, excitatory GABA events co-occur with glutamate to enhance intracellular Ca^2+^ increase. Here again, this excitatory activity plays a trophic role in dendritic growth, as described in neonatal hippocampal neurons^39^. Transient excitatory trophic effects were also described in postnatal CA3 pyramidal neurons in mouse^34^ and rat^40^. All of these trophic effects of GABA have been ascribed to dGPSPs being fully excitatory.

However, in immature neural networks that involve exclusively functional excitatory synapses (*i.e*., Ach, Glu, GABA and glycine), a crucial question arises concerning the termination of spontaneous activity and the avoidance of deleterious overexcitation and excitotoxicity. We demonstrate that dGPSPs may play a key role in both controlling the termination of spontaneous activity waves and preventing overactivity. Indeed, during an episode of spontaneous activity, the dGPSP discharge frequency increases. This increase would naturally lead to a switch from excitation to inhibition (see Fig. 7B,C) and to the cessation of the episode. This mechanism, combined with other homeostatic mechanisms, *i.e*., the opening of the activity-dependent K^+^-channel or Ca^2+^-activated K^+^ channels, and synaptic depression^41^,^42^,^43^ may contribute to auto-limited excitation produced by dGPSPs. Moreover, in addition to controlling their own activity, dGPSPs also overcome other excitatory synaptic outcomes (see Fig. 9). In the absence of dGPSPs, these excitatory synapses would lead to detrimental overexcitation.

Properties of dGPSPs can also be modulated by the presence of intrinsic conductances, such as low-voltage-activated Ca^2+^ currents (I_t_) and hyperpolarization-activated, mixed cationic currents (I_h_)^44^, which support transient depolarizing events. When present, such intrinsic conductances would favor excitatory effects at the onset of dGPSP trains. Interestingly, because the I_h_ current is expressed to a greater degree in embryonic spinal MNs (personal observations) than in post-natal MNs^45^, the switch from excitation to inhibition during dGPSP trains is likely reinforced at early stages. Neonatal spinal MNs also express persistent sodium currents (I_NaP_)^46^ that would enhance the excitatory component of dGPSP trains. In addition, short-term plasticity may also impact the effect of dGPSP trains. As was shown in post-natal dorsal horn neurons^47^, evoked-GABA synaptic events exhibit frequency-dependent short-term facilitation and depression. However, evidence for a major role of such plasticity was not found during the sGPSP train paradigm used here.

### Functional relevance of dGPSPs in the mature CNS

Although dGPSPs are transient throughout development, they persist during adulthood in certain neurons^48^,^49^. In most cases, dGPSP-induced depolarization is small (a few millivolts)^13^,^14^,^40^ and exerts primarily inhibitory effects *via* a shunting mechanism. Interestingly, the excitatory effect of GPSPs relies on the identity of the presynaptic interneuron as demonstrated in cortical pyramidal neurons, in which chandelier cells evoke excitatory dGPSPs on the axon initial segment (AIS). However, GPSPs originating from basket cells exert inhibitory action on somatic and dendritic perisomatic compartments^50^. These differential effects of GABA inputs are linked to E_Cl_ values that vary among neuron compartments^16^. This type of organization has not been described for MNs.

Jean-Xavier *et al*. have used ventral funiculus (VF) electrical stimulations to evoke IPSPs in postnatal rat spinal MNs. To mimic the effect of physiological IPSPs, simulations were performed using a large g_Clp_ (120 nS), indicating that the VF stimulation massively recruited inhibitory synapses to MNs. Consequently, these authors primarily observed inhibitory effects. In the present study, we explored dGPSPs sustained by smaller g_Clp_ values (1–7 nS) to assess the effect of recruiting few synapses, leading to a nearly complete excitatory effect (Fig. 3F). Therefore, our study demonstrates that the g_Clp_ value controls the extent and magnitude of the inhibitory domain in dGPSPs.

In mature (P25) rat cerebellar interneurons^51^, excitatory and inhibitory dGPSPs coexist because E_GABA_ is depolarized but close to the mean membrane potential. Therefore, dGPSPs buffer the mean firing rate of targeted interneurons. In mature (P26) mouse interneurons of the dorsal cochlear nucleus^52^ and in adult rat dentate gyrus granule cells^5^, E_Cl_ is >10 mV more depolarized than E_Rest_. However, the shunting effect of dGPSPs largely prevails if the membrane potential remains close to E_Rest_. In P21-28 layer 5 pyramidal neurons from the somatosensory cortex^1^, neurons exhibit large hyperpolarizing oscillations, such as during periodic “down” states. In this case, dGPSPs become largely depolarizing and excitatory and therefore may enhance the excitability of other depolarizing inputs.

Depolarizing GPSPs may also play a role in tuning mechanisms. For example, in mature cortical pyramidal neurons, dGPSPs favor late occurring spikes (during the excitatory portion of the dGPSPs) or early occurring block spikes (during the inhibitory portion of the dGPSPs)^1^. Additionally, rapid inhibitory neurotransmission in which GABA/glycine is depolarizing plays a prominent role in encoding the auditory cues that are involved in sound localization in the vertebrate auditory brainstem^10^. In this system, acoustically driven GABA- and glycine-mediated inputs have been described as inhibitory (shunting inhibition).

### Switch from excitation to inhibition during tonic GABA/glycine release

A large part of the literature is devoted to analyzing the depolarizing effects that are elicited by the tonic activation of GABA/glycine receptors. The bath application of increasing GABA concentrations led to a switch from excitation to inhibition in hippocampal adult interneurons^11^,^53^,^54^. Under these conditions, by inducing a persistent increase in Cl^−^ conductance, the tonic shunting inhibitory effect eventually overpowered depolarizing excitatory effects.

Our results addressed this point and extended these previous findings by demonstrating a similar behavior with a switch from activation to inhibition when GABA/glycine receptors are synaptically activated at a high frequency, *i.e*., the role that is played by increasing g_Clp_ (g_GABA_ in tonic GABA studies) was transposed in the frequency domain. The frequencies that are explored in the present simulations are high but may represent the concomitant discharge of several GABA/glycine presynaptic afferents. Such global frequencies of IPSPs have been recorded in neonatal spinal MNs during locomotor-like activity^55^ and in adult hippocampal CA1 pyramidal neurons^56^. These mechanisms may be responsible for the occurrence of alternations between left and right spinal locomotor networks during prenatal stages^57^,^58^.

Our findings provide a precise analysis of the dGPSP outcome based on the chloride conductance value, in relation to the chloride ion driving force and inhibitory/excitatory time course. Collectively, these findings shed new light on the dynamics of the inhibitory/excitatory interplay operating in dGPSPs. As demonstrated herein, this analysis provides a comprehensive quantitative background that is necessary for the prediction of the outcome of the entire variety of previously reported dGPSPs.

## Materials and Methods

### Animals and spinal cord preparation

Pregnant adult OF1 mice (Charles River Laboratories, l’Arbresle, France) were maintained and sacrificed in accordance with the European Communities Council Directive (86 ⁄609 ⁄ EEC). The local Ethical Committee for Animal Research (CEEA50) of the University of Bordeaux approved the experiments and the methods were conducted in accordance with the approved guidelines. The experiments were conducted on embryos at E13.5 and E17.5 of either sex. The embryos were surgically removed from pregnant mice that were sacrificed by cervical dislocation and submerged in cold (6–8 °C) artificial cerebrospinal fluid (aCSF) containing (in mM) 114.5 NaCl, 3 KCl, 2 CaCl_2_·2H_2_O, 1 MgCl_2_·6H_2_O, 25 NaHCO_3_, 1 NaH_2_PO4·H_2_O, and 11 D-glucose, pH 7.4 (296 mosmol·L^−1^) oxygenated with a 95% O_2_/5% CO_2_ mixture. The embryos were decapitated, and the brainstem-spinal cord preparation was dissected. The spinal cord was dorsally opened, and the meninges were removed. The preparation was placed in a recording chamber (ventral side up), maintained opened under a nylon mesh, and superfused (~1.5 mL·min^−1^) with the oxygenated aCSF. All of the experiments were performed at a constant temperature (30 °C). For each brainstem-spinal cord preparation, we recorded only 1 to 3 MNs. Hence, fifteen E13.5 MNs (13 embryos from 12 different pregnant mice) and thirteen E17.5 MNs (8 embryos from 6 different pregnant mice) were analysed from a total of 18 pregnant mice.

### Whole-cell patch-clamp recordings and isoguvacine local pressure ejection

We performed whole-cell patch-clamp recordings from lumbar spinal MNs that were identified according to their morphological features (pear-shaped large cell body), their disposition in the ventral column^17^ and their capacitance c_m_ (2- to 4-fold larger than interneurons). A Zeiss Axioskop 2 FS Plus microscope (Marly le Roi, France) equipped with differential interference contrast and a CCD camera (Zeiss AxioCam MRm, Marly le Roi, France) was used to visualize the MNs. Patch-clamp electrodes and isoguvacine-containing pipettes were pulled from thin-walled single-filamented borosilicate glass (1.5-mm outer diameter, Harvard Apparatus, Les Ulis, France) using a two-stage vertical microelectrode puller (PP-830, Narishige, Tokyo, Japan). Patch electrode resistances, ranging from 3 to 5 MΩ, were filled with intracellular medium containing (in mM) 107 K gluconate, 23 KCl, 10 HEPES, 10 EGTA, 5 NaCl, 2 Mg·ATP and 1 CaCl_2_·2H_2_O, pH 7.4 (297 mosmol·L^−1^), for E13.5 and (in mM) 124 K gluconate, 5 KCl, 10 HEPES, 10 EGTA, 6 NaCl, 2 Mg·ATP and 1 CaCl_2_·2H_2_O, pH 7.4 (297 mosmol·L^−1^), for E17.5, to fit physiological E_Cl_ values as measured in E13.5 and E17.5 MNs^17^ or to intermediate E_Cl_ values. The highly specific GABA_A_R agonist isoguvacine (1 mM) was locally applied *via* a glass pipette in the vicinity of MN soma by pressure ejection (50 ms; ∼1 psi) using a Picospritzer II (General Valve, Fairfield, NJ, USA) driven by a programmable Master 8 Stimulator/Pulse Generator (Master-8, A.M.P.I. Jerusalem, Israel). The amount of ejected isoguvacine was adjusted to evoke a response of the desired amplitude (20–30% of the saturating effect). Motorized micromanipulators (Luigs & Neumann, Ratingen, Germany) were used to position a patch-clamp electrode on and an isoguvacine ejecting pipette close to a visually identified MN (Suppl. Fig. 2A).

All of the recordings were performed using an Axon MultiClamp 700B amplifier (Molecular Devices, Sunnyvale, CA, USA). The data were low-pass filtered (2 kHz) and acquired at 20 kHz on a computer *via* an analog-to-digital converter (Digidata 1322A, Molecular Devices) and data acquisition software (pCLAMP 10.3, Molecular Devices). The voltage compensation details are summarized in the Supplemental Experimental Procedures.

### Estimation of the inhibitory/excitatory effects of the MN response to isoguvacine pressure ejection

Depolarizing threshold current pulses (10-ms duration) were injected through the recording patch-clamp pipette in current-clamp mode to evoke one action potential in half of the trails. These pulses were concomitantly delivered to isoguvacine puffs. The quantification of the inhibitory *versus* excitatory effects of the GABA_A_R agonist isoguvacine was investigated by the alteration of the probability of spike triggering. The following protocol was used: the threshold current pulse was adjusted to obtain a spike triggering probability of ~0.5 (trial) (Suppl. Fig. 3B). A fifth current pulse was generated to estimate the effect of the isoguvacine puff (test). The delay between the fifth current pulse (test) and the onset of the isoguvacine puff was gradually modified in successive assays from −20 ms (before the puff) to 80 ms (end of the test window during the puff) by 20 ms steps. The percentage of spikes that were triggered by depolarizing current pulses along the isoguvacine puff divided by the percentage of spikes that were triggered before the puff defined R_GABA_ (for additional details, see Supplemental Experimental Procedures).

### Immunohistochemistry

Four E13.5 MNs and four E17.5 MNs were stained during the whole-cell recordings with pipettes containing neurobiotin (0.4%, CliniSciences, Montrouge, France) diluted in the intracellular medium. After the recording session, during which the MNs were injected with neurobiotin, the entire brainstem-spinal cord preparation was fixed in 4% paraformaldehyde (PFA) for 2 h at room temperature. The preparation was then rinsed three times with 0.1 M phosphate-buffered saline (PBS) and incubated with streptavidin-Cy3 (1:400, Life Technologies SAS, Saint-Aubin, France) overnight at 4 °C in 0.1 M PBS containing 0.2% bovine serum albumin (Sigma Aldrich, St. Louis, MO, USA) and 0.3% Triton X-100 (Sigma). To confirm that the injected neurons were MNs, a mouse monoclonal antibody against Islet-1/2 (1/100, Developmental Studies Hybridoma Bank), a marker of developing MNs^59^, was added to the incubation medium (data not shown). The preparation was incubated with Alexa Fluor 488 goat anti-mouse IgG (H + L) (1:400, Life Technologies SAS) for 2 h at room temperature, abundantly rinsed in 0.1 M PBS, and finally mounted with anti-fade reagent (Fluoromount, Electron Microscopy Sciences, PA, USA). Figure 1A,B illustrate representative neurobiotin-injected E13.5 and E17.5 MNs.

### Confocal microscopy and reconstruction procedures

Spinal MNs were imaged with a BX51 Olympus FluoView 500 confocal microscope (Olympus France, Rungis). A three-dimensional (3D) reconstruction of E13.5 and E17.5 MNs was performed using the *Neurolucida* confocal module (MBF Bioscience Inc., Williston, VT, USA) following shrinkage correction (see Supplemental Experimental Procedures). Morphometric parameters characterizing morphological and topological features of MN dendritic arborization were harvested using the *Neurolucida Explorer* software package (MBF Bioscience Inc.). These parameters were used to define the canonical E13.5 and E17.7 MNs that were used in the simulations (see Fig. 1A_2_–B_2_).

### Computer simulations

The synaptic effects of GABA/glycine on the E13.5 and E17.5 MNs were simulated using a compartment model that was elaborated with the program NEURON 7.3^60^. Two simulated neurons were constructed: an E13.5 MN (Fig. 1A_2_) and an E17.5 MN (Fig. 1B2). Both of the neurons were constructed according to canonical MNs that were designed from the average topology and morphology of four E13.5 and four E17.5 MNs, respectively. The canonical E13.5 MN was composed of a cell body (12.17 μm in diameter), 6 main dendrites and an axon (Suppl. Table 1 in the Supplemental Information). The E17.5 MN was composed of a cell body (17.27 μm in diameter), 9 main dendrites and an axon (Suppl. Table 2 in the Supplemental Information).

Inhibitory and excitatory synaptic inputs were inserted into the somatic MN compartment and not on proximal or distal dendrites. The spatial effects of dGPSPs have previously been described in the literature^13^ and were not studied in the present simulations. Equations on the channel properties and GABA_A_R synaptic activation are summarized in the Supplemental Experimental Procedures. The specific membrane resistance was adjusted to fit the R_in_ of the corresponding real neurons. The density of the Na^+^ channels on the axonal initial segment was adjusted to fit E_Thr_.

The time course of inhibitory and excitatory effects of dGPSPs was quantified by determining the threshold current that is required to elicit a spike during a 10-ms depolarizing current pulse. A recursive algorithm was used to approach the threshold current with a precision of 10^−6^ nA. This procedure was repeated throughout the dGPSP time course, every 10 ms, beginning 80 ms prior to onset and ending 300 ms following onset. To increase the precision of the curve at the onset of the simulated dGPSP, the threshold was estimated every 1 ms. Consequently, a curve that represents the threshold as a function of time was obtained. These curves were generated at various E_Rest_ values (typically from −85 mV to −65 mV). Each of the threshold current curves was used to construct a representation of the relative inhibitory/excitatory effect of dGPSPs over time, which is expressed as a percentage of the threshold current measured prior to onset of the synaptic event. For a given value of E_Cl_, the curves representing the time course of the inhibitory/excitatory effect during a dGPSP at different E_Rest_ values were combined into a 2D-level map (inhibitory excitatory time course; IETC maps) with the X-axis representing time (ranging from −80 ms to 300 ms; 0 representing the onset of a dGPSP) and the Y-axis representing the E_Rest_ of the neuron model. Contours are disposed at every 1% relative change in the threshold current. The IETC maps were colored in blue and red for inhibitory and excitatory effects, respectively (for additional details on the color code, see Supplemental Experimental Procedures). This analysis was performed using the free software R version 3.0.2 (http://www.r-project.org).

### Statistical analysis

For the statistical analysis, we used either a non-parametric Mann-Whitney U test or one-way ANOVA test followed by Tukey’s multiple comparison *post-hoc* test (GraphPad Prism 6 software, La Jolla, CA, USA). The results are expressed as the means ± SEM.

## Additional Information

**How to cite this article**: Branchereau, P. *et al*. Depolarizing GABA/glycine synaptic events switch from excitation to inhibition during frequency increases. *Sci. Rep*. **6**, 21753; doi: 10.1038/srep21753 (2016).

## Supplementary Material

Supplementary Information

## Figures and Tables

**Table 1:** Physiological values measured in spinal MNs according to different configurations.

Developmental Stage	E13.5	E13.5		E17.5	E17.5
Intracellular Medium Type	E13.5-like	E17.5-like		E17.5-like	E13.5
		Low g_Clp_	High g_Clp_		
R_GABA_# Measurements	(9)	(5)	(5)	(10)	(10)
E_Rest_ (mV)	−73.7 ± 1.9 (6)	−73.2 ± 3.8 (4)	−72.6 ± 1.2 (3)	−85.8 ± 0.8 (7)	−77.1 ± 1.7 (6)
E_Cl_ (mV)	−44.0 ± 1.9 (6)	−65.9 ± 2.1 (4)	−67.8 ± 1.0 (3)	−82.0 ± 2.3 (5)	−55.3 ± 3.8 (5)
[Cl^−^]_i_ (mM)	23.1 ± 1.8 (6)	9.9 ± 0.8 (4)	9.2 ± 0.3 (3)	5.4 ± 0.4 (5)	15.5 ± 1.9 (5)
Intra-pipette [Cl^−^] (mM)	30	13	13	13	30
g_Clp_ (nS)	1.6 ± 0.2 (6)	2.3 ± 0.1 (4)	3.6 ± 0.4 (3)	5.2. ± 1.4 (4)	4.1 ± 0.8 (4)

Low g_Clp_ < 2.6 nS and high g_Clp_ > 2.6 nS. Numbers in parentheses indicate experiments in which measures were performed. R_GABA_ was assessed twice in certain experiments.

**Table 2:** Physiological values measured in E13.5 spinal MNs during VF stimulation experiments.

Developmental Stage	E13.5
Intracellular Medium Type	E13.5-like
E_Rest_ (mV)	−69.7 ± 1.8 (5)
R_in_	790 ± 43 (5)
C_m_ (MΩ)	37.2 ± 1.9 (5)
E_Cl_ (mV)**	−48.5 ± 2.5 (5)
[Cl^−^]_i_ (mM)	19.6 ± 1.6 (5)
Intra-pipette [Cl^−^] (mM)	30
g_Clp_ (nS)	2.4 ± 1.2 (5)

^**^E_Cl_ was estimated from dGPSPs evoked by VF stimulation.

## References

[b1] Gulledge A. T. & Stuart G. J. Excitatory actions of GABA in the cortex. Neuron 37, 299–309 (2003). 12546824 10.1016/s0896-6273(02)01146-7

[b2] Alger B. E. & Nicoll R. A. Pharmacological evidence for two kinds of GABA receptor on rat hippocampal pyramidal cells studied *in vitro*. J Physiol 328, 125–141 (1982). 7131310 10.1113/jphysiol.1982.sp014256 PMC1225650

[b3] Michelson H. B. & Wong R. K. Excitatory synaptic responses mediated by GABAA receptors in the hippocampus. Science 253, 1420–1423 (1991). 1654594 10.1126/science.1654594

[b4] Misgeld U., Wagner A. & Ohno T. Depolarizing IPSPs and Depolarization by GABA of rat neostriatum cells *in vitro*. Exp Brain Res 45, 108–114 (1982). 7056316 10.1007/BF00235769

[b5] Staley K. J. & Mody I. Shunting of excitatory input to dentate gyrus granule cells by a depolarizing GABAA receptor-mediated postsynaptic conductance. J Neurophysiol 68, 197–212 (1992). 1381418 10.1152/jn.1992.68.1.197

[b6] Cherubini E., Gaiarsa J. L. & Ben-Ari Y. GABA: an excitatory transmitter in early postnatal life. Trends Neurosci 14, 515–519 (1991). 1726341 10.1016/0166-2236(91)90003-d

[b7] Ben-Ari Y. Developing networks play a similar melody. Trends Neurosci 24, 353–360 (2001). 11356508 10.1016/s0166-2236(00)01813-0

[b8] Hanson M. G. & Landmesser L. T. Characterization of the circuits that generate spontaneous episodes of activity in the early embryonic mouse spinal cord. J Neurosci 23, 587–600 (2003). 12533619 10.1523/JNEUROSCI.23-02-00587.2003 PMC6741864

[b9] Czarnecki A. . Acetylcholine controls GABA-, glutamate-, and glycine-dependent giant depolarizing potentials that govern spontaneous motoneuron activity at the onset of synaptogenesis in the mouse embryonic spinal cord. J Neurosci 34, 6389–6404 (2014). 24790209 10.1523/JNEUROSCI.2664-13.2014 PMC6608101

[b10] Nerlich J., Keine C., Rubsamen R., Burger R. M. & Milenkovic I. Activity-dependent modulation of inhibitory synaptic kinetics in the cochlear nucleus. Front Neural Circuits 8, 145 (2014). 25565972 10.3389/fncir.2014.00145 PMC4274880

[b11] Song I., Savtchenko L. & Semyanov A. Tonic excitation or inhibition is set by GABA(A) conductance in hippocampal interneurons. Nat Commun 2, 376 (2011). 21730957 10.1038/ncomms1377 PMC3144593

[b12] Kolbaev S. N., Achilles K., Luhmann H. J. & Kilb W. Effect of depolarizing GABA(A)-mediated membrane responses on excitability of Cajal-Retzius cells in the immature rat neocortex. J Neurophysiol 106, 2034–2044 (2011). 21775719 10.1152/jn.00699.2010

[b13] Jean-Xavier C., Mentis G. Z., O’Donovan M. J., Cattaert D. & Vinay L. Dual personality of GABA/glycine-mediated depolarizations in immature spinal cord. Proc Natl Acad Sci USA 104, 11477–11482 (2007). 17592145 10.1073/pnas.0704832104 PMC2040923

[b14] Gao X. B., Chen G. & van den Pol A. N. GABA-dependent firing of glutamate-evoked action potentials at AMPA/kainate receptors in developing hypothalamic neurons. J Neurophysiol 79, 716–726 (1998). 9463435 10.1152/jn.1998.79.2.716

[b15] Farrant M. & Kaila K. The cellular, molecular and ionic basis of GABA(A) receptor signalling. Prog Brain Res 160, 59–87 (2007). 17499109 10.1016/S0079-6123(06)60005-8

[b16] Khirug S. . GABAergic depolarization of the axon initial segment in cortical principal neurons is caused by the Na-K-2Cl cotransporter NKCC1. J Neurosci 28, 4635–4639 (2008). 18448640 10.1523/JNEUROSCI.0908-08.2008 PMC6670448

[b17] Delpy A., Allain A. E., Meyrand P. & Branchereau P. NKCC1 cotransporter inactivation underlies embryonic development of chloride-mediated inhibition in mouse spinal motoneuron. J Physiol 586, 1059–1075 (2008). 18096599 10.1113/jphysiol.2007.146993 PMC2375629

[b18] Rivera C. . The K^+^/Cl^−^ co-transporter KCC2 renders GABA hyperpolarizing during neuronal maturation. Nature 397, 251–255 (1999). 9930699 10.1038/16697

[b19] Payne J. A., Rivera C., Voipio J. & Kaila K. Cation-chloride co-transporters in neuronal communication, development and trauma. Trends Neurosci 26, 199–206 (2003). 12689771 10.1016/S0166-2236(03)00068-7

[b20] Kaila K., Lamsa K., Smirnov S., Taira T. & Voipio J. Long-lasting GABA-mediated depolarization evoked by high-frequency stimulation in pyramidal neurons of rat hippocampal slice is attributable to a network-driven, bicarbonate-dependent K+ transient. J Neurosci 17, 7662–7672 (1997). 9315888 10.1523/JNEUROSCI.17-20-07662.1997 PMC6793904

[b21] Kaila K., Price T. J., Payne J. A., Puskarjov M. & Voipio J. Cation-chloride cotransporters in neuronal development, plasticity and disease. Nat Rev Neurosci 15, 637–654 (2014). 25234263 10.1038/nrn3819 PMC4294553

[b22] Bormann J., Hamill O. P. & Sakmann B. Mechanism of anion permeation through channels gated by glycine and gamma-aminobutyric acid in mouse cultured spinal neurones. J Physiol 385, 243–286 (1987). 2443667 10.1113/jphysiol.1987.sp016493 PMC1192346

[b23] Brocard F., Vinay L. & Clarac F. Gradual development of the ventral funiculus input to lumbar motoneurons in the neonatal rat. Neuroscience 90, 1543–1554 (1999). 10338319 10.1016/s0306-4522(98)00550-8

[b24] Okun M. & Lampl I. Instantaneous correlation of excitation and inhibition during ongoing and sensory-evoked activities. Nat Neurosci 11, 535–537 (2008). 18376400 10.1038/nn.2105

[b25] Destexhe A., Rudolph M., Fellous J. M. & Sejnowski T. J. Fluctuating synaptic conductances recreate *in vivo*-like activity in neocortical neurons. Neuroscience 107, 13–24 (2001). 11744242 10.1016/s0306-4522(01)00344-x PMC3320220

[b26] Le Bon-Jego M., Cattaert D. & Pearlstein E. Serotonin enhances the resistance reflex of the locomotor network of the crayfish through multiple modulatory effects that act cooperatively. J Neurosci 24, 398–411 (2004). 14724238 10.1523/JNEUROSCI.4032-03.2004 PMC6730000

[b27] Boulenguez P. . Down-regulation of the potassium-chloride cotransporter KCC2 contributes to spasticity after spinal cord injury. Nat Med 16, 302–307 (2010). 20190766 10.1038/nm.2107

[b28] Cordero-Erausquin M., Coull J. A., Boudreau D., Rolland M. & De Koninck Y. Differential maturation of GABA action and anion reversal potential in spinal lamina I neurons: impact of chloride extrusion capacity. J Neurosci 25, 9613–9623 (2005). 16237166 10.1523/JNEUROSCI.1488-05.2005 PMC6725724

[b29] Baccei M. L. & Fitzgerald M. Development of GABAergic and glycinergic transmission in the neonatal rat dorsal horn. J Neurosci 24, 4749–4757 (2004). 15152035 10.1523/JNEUROSCI.5211-03.2004 PMC6729459

[b30] Singer J. H., Talley E. M., Bayliss D. A. & Berger A. J. Development of glycinergic synaptic transmission to rat brain stem motoneurons. J Neurophysiol 80, 2608–2620 (1998). 9819267 10.1152/jn.1998.80.5.2608

[b31] Ritter B. & Zhang W. Early postnatal maturation of GABAA-mediated inhibition in the brainstem respiratory rhythm-generating network of the mouse. Eur J Neurosci 12, 2975–2984 (2000). 10971638 10.1046/j.1460-9568.2000.00152.x

[b32] Balakrishnan V. . Expression and function of chloride transporters during development of inhibitory neurotransmission in the auditory brainstem. J Neurosci 23, 4134–4145 (2003). 12764101 10.1523/JNEUROSCI.23-10-04134.2003 PMC6741087

[b33] Ehrlich I., Lohrke S. & Friauf E. Shift from depolarizing to hyperpolarizing glycine action in rat auditory neurones is due to age-dependent Cl- regulation. J Physiol 520 Pt 1, 121–137 (1999). 10517806 10.1111/j.1469-7793.1999.00121.x PMC2269580

[b34] Banke T. G. & McBain C. J. GABAergic input onto CA3 hippocampal interneurons remains shunting throughout development. J Neurosci 26, 11720–11725 (2006). 17093093 10.1523/JNEUROSCI.2887-06.2006 PMC6674795

[b35] Khazipov R. . Developmental changes in GABAergic actions and seizure susceptibility in the rat hippocampus. Eur J Neurosci 19, 590–600 (2004). 14984409 10.1111/j.0953-816x.2003.03152.x

[b36] Ben-Ari Y., Cherubini E., Corradetti R. & Gaiarsa J. L. Giant synaptic potentials in immature rat CA3 hippocampal neurones. J Physiol 416, 303–325 (1989). 2575165 10.1113/jphysiol.1989.sp017762 PMC1189216

[b37] Owens D. F. & Kriegstein A. R. Is there more to GABA than synaptic inhibition? Nat Rev Neurosci 3, 715–727 (2002). 12209120 10.1038/nrn919

[b38] Hanson M. G. & Landmesser L. T. Normal patterns of spontaneous activity are required for correct motor axon guidance and the expression of specific guidance molecules. Neuron 43, 687–701 (2004). 15339650 10.1016/j.neuron.2004.08.018

[b39] Leinekugel X., Tseeb V., Ben-Ari Y. & Bregestovski P. Synaptic GABAA activation induces Ca^2+^ rise in pyramidal cells and interneurons from rat neonatal hippocampal slices. J Physiol 487 (**Pt 2**), 319–329 (1995). 8558466 10.1113/jphysiol.1995.sp020882 PMC1156575

[b40] Tyzio R. . Postnatal changes in somatic gamma-aminobutyric acid signalling in the rat hippocampus. Eur J Neurosci 27, 2515–2528 (2008). 18547241 10.1111/j.1460-9568.2008.06234.x

[b41] Tabak J., Rinzel J. & O’Donovan M. J. The role of activity-dependent network depression in the expression and self-regulation of spontaneous activity in the developing spinal cord. J Neurosci 21, 8966–8978 (2001). 11698607 10.1523/JNEUROSCI.21-22-08966.2001 PMC6762295

[b42] Chub N. & O’Donovan M. J. Post-episode depression of GABAergic transmission in spinal neurons of the chick embryo. J Neurophysiol 85, 2166–2176 (2001). 11353031 10.1152/jn.2001.85.5.2166

[b43] Chub N., Mentis G. Z., O’Donovan M. J. & Chloride-sensitive M. E. Q. fluorescence in chick embryo motoneurons following manipulations of chloride and during spontaneous network activity. J Neurophysiol 95, 323–330 (2006). 16192339 10.1152/jn.00162.2005

[b44] Robinson R. B. & Siegelbaum S. A. Hyperpolarization-activated cation currents: from molecules to physiological function. Annu Rev Physiol 65, 453–480 (2003). 12471170 10.1146/annurev.physiol.65.092101.142734

[b45] Bertrand S. & Cazalets J. R. Postinhibitory rebound during locomotor-like activity in neonatal rat motoneurons *in vitro*. J Neurophysiol 79, 342–351 (1998). 9425203 10.1152/jn.1998.79.1.342

[b46] Bouhadfane M., Tazerart S., Moqrich A., Vinay L. & Brocard F. Sodium-mediated plateau potentials in lumbar motoneurons of neonatal rats. J Neurosci 33, 15626–15641 (2013). 24068829 10.1523/JNEUROSCI.1483-13.2013 PMC6618457

[b47] Ingram R. A., Fitzgerald M. & Baccei M. L. Developmental changes in the fidelity and short-term plasticity of GABAergic synapses in the neonatal rat dorsal horn. J Neurophysiol 99, 3144–3150 (2008). 18400957 10.1152/jn.01342.2007

[b48] Ben-Ari Y., Tseeb V., Raggozzino D., Khazipov R. & Gaiarsa J. L. gamma-Aminobutyric acid (GABA): a fast excitatory transmitter which may regulate the development of hippocampal neurones in early postnatal life. Prog Brain Res 102, 261–273 (1994). 7800817 10.1016/S0079-6123(08)60545-2

[b49] Staley K. J., Soldo B. L. & Proctor W. R. Ionic mechanisms of neuronal excitation by inhibitory GABAA receptors. Science 269, 977–981 (1995). 7638623 10.1126/science.7638623

[b50] Szabadics J. . Excitatory effect of GABAergic axo-axonic cells in cortical microcircuits. Science 311, 233–235 (2006). 16410524 10.1126/science.1121325

[b51] Chavas J. & Marty A. Coexistence of excitatory and inhibitory GABA synapses in the cerebellar interneuron network. J Neurosci 23, 2019–2031 (2003). 12657660 10.1523/JNEUROSCI.23-06-02019.2003 PMC6742031

[b52] Golding N. L. & Oertel D. Context-dependent synaptic action of glycinergic and GABAergic inputs in the dorsal cochlear nucleus. J Neurosci 16, 2208–2219 (1996). 8601801 10.1523/JNEUROSCI.16-07-02208.1996 PMC6578533

[b53] Pavlov I. . Tonic GABAA conductance bidirectionally controls interneuron firing pattern and synchronization in the CA3 hippocampal network. Proc Natl Acad Sci USA 111, 504–509 (2014). 24344272 10.1073/pnas.1308388110 PMC3890854

[b54] Tang Z. Q., Dinh E. H., Shi W. & Lu Y. Ambient GABA-activated tonic inhibition sharpens auditory coincidence detection via a depolarizing shunting mechanism. J Neurosci 31, 6121–6131 (2011). 21508237 10.1523/JNEUROSCI.4733-10.2011 PMC3090224

[b55] Kiehn O., Johnson B. R. & Raastad M. Plateau properties in mammalian spinal interneurons during transmitter-induced locomotor activity. Neuroscience 75, 263–273 (1996). 8923540 10.1016/0306-4522(96)00250-3

[b56] Ylinen A. . Sharp wave-associated high-frequency oscillation (200 Hz) in the intact hippocampus: network and intracellular mechanisms. J Neurosci 15, 30–46 (1995). 7823136 10.1523/JNEUROSCI.15-01-00030.1995 PMC6578299

[b57] Branchereau P. . Development of lumbar rhythmic networks: from embryonic to neonate locomotor-like patterns in the mouse. Brain Res Bull 53, 711–718 (2000). 11165805 10.1016/s0361-9230(00)00403-2

[b58] Nishimaru H. & Kudo N. Formation of the central pattern generator for locomotion in the rat and mouse. Brain Res Bull 53, 661–669 (2000). 11165801 10.1016/s0361-9230(00)00399-3

[b59] Ericson J., Thor S., Edlund T., Jessell T. M. & Yamada T. Early stages of motor neuron differentiation revealed by expression of homeobox gene Islet-1. Science 256, 1555–1560 (1992). 1350865 10.1126/science.1350865

[b60] Hines M. L. & Carnevale N. T. The NEURON simulation environment. Neural computation 9, 1179–1209 (1997). 9248061 10.1162/neco.1997.9.6.1179

