# Supplementary material for: Depolarizing GABA/glycine synaptic events switch from excitation to inhibition during frequency increases
Source: Sci Rep. 2016 Feb 25;6:21753. doi: 10.1038/srep21753 (PMC4766471; doi:10.1038/srep21753)
Supplement: Supplementary Information [file srep21753-s1.pdf]

Depolarizing GABA/glycine synaptic events switch from excitation to inhibition during frequency increases

Pascal Branchereau[1]*, Daniel Cattaert[1]*, Alain Delpy[1], Anne-Emilie Allain[1], Elodie Martin[1] and Pierre Meyrand[2]

[1]Univ. Bordeaux, INCIA, UMR 5287, F-33615 Pessac, France. CNRS, INCIA, UMR 5287, F-33615 Pessac, France.
[2]Present Address: Univ. Bordeaux, IMN, UMR 5293, F-33615 Pessac, France. CNRS, IMN, UMR 5293, F-33615 Pessac, France.
*These authors contributed equally to this work.

Supplementary Information

Supplementary Figure Legends

Supplementary Figure 1: **A**, Depolarizing GPSPs that were produced in E13.5 (**A1**) and E17.5 (**A2**) neuron models using an alpha-synapse formalism with a time constant of 20 ms and various synaptic conductances (g_{Clp}). The curves of peak amplitude against conductance (g_{Clp}) for the E13.5 (green) and E17.5 (red) neuron models are presented in **A3**. **B**, Time course of the synaptic conductance (g_{Cl}, red) and membrane potential (*Em*, green) during a simulated dGPSP in the E13.5 neuron model with g_{Clp} = 3.5 nS.

Supplementary Figure 2: Comparison of the time constants during the recovery phase following a current pulse and following an isoguvacine puff in the E13.5 and E17.5 MNs. A, Disposition of the patch-clamp recording and isoguvacine-containing pipettes. **A1**. Phase contrast photomicrography. **A2**, Schematic drawing of **A1**. **B1**, Response of an E13.5 MN to a 15-pA depolarizing current pulse. **B2**, Response of the same E13.5 MN to an isoguvacine puff. **B3**, Fitting (one exponential decay) of the recovery phase of the recordings that are illustrated in **A1** (blue curve) and **A2** (orange curve). **C1-3**, The same disposition as in **B1-3** but in an E17.5 MN. i: 95-pA current pulse in **B1**.

Supplementary Figure 3: Experimental protocol that was developed to estimate the excitatory *versus* inhibitory component of dGPSPs. A, Estimation of E_{Cl}. **A1**, Current responses of an E13.5 MN to an isoguvacine puff at a series of holding potentials in voltage-clamp mode. **A2**, Amplitude of the responses that were obtained in **A1** against the holding potential E_m. The slope of the regression line yields the peak conductance (g_{Clp}) as activated by the isoguvacine puff. This line intercepts the zero current at the E_{Cl} value (-41 mV in the illustrated example). **B**, Original method that was developed to quantify the inhibitory and excitatory isoguvacine effects, as expressed by R_{GABA}. This parameter is a ratio and is calculated

as the percentage of spikes that were triggered by depolarizing current pulses during the isoguvacine puff divided by the percentage of spikes that were triggered during control trials preceding the puff (at least 4 series as illustrated). **B1-B2**, Series of 4 trials of depolarizing pulses preceded the puff with a fifth pulse 20 ms prior to puff onset (**B1**) or 20 ms following puff onset (**B2**) (see inset in **B3**). The illustrated experiments were performed on an E13.5 MN at E_{Rest} = -71 mV, and the resultant R_{GABA} values are shown in **B3**. The traces in **B1-2** are right shifted for clarity.

Supplementary Figure 4: Alteration of E_{Cl} during $GABA_A$/glycine receptor activation. In an E17.5 MN, E_{Cl} was measured before (**A1**) and after (**A2**) a series of isoguvacine puffs (1 Hz for 1 min). Following the repetitive activation of $GABA_AR$, the dGPSP amplitude decreased from 4.7 mV to 2.0 mV (**B1**), whereas the E_{Cl} shifted from -77 mV to -64 mV (**B2**).

Supplementary Figure 5: Fitting of the decay phase of the synaptic conductance. To calculate the steady-state conductance that was reached during dGPSP trains (see text for details), the decay phase was fitted to a single exponential decay curve. The value at t = 0 yields $K \times g_{Max}$ as used in the equation (1) providing the limit of summated conductances ($g_{Peaklim}$):

$$g_{Peaklim} = g_{Max} + K \times g_{Max} \times \left(\frac{e^{\frac{-1}{N\tau}}}{1 - e^{\frac{-1}{N\tau}}} \right) \quad (1)$$

in which N is the dGPSP train frequency, τ is the time constant of dGPSP decay, and K is the coefficient that is used to assimilate the dGPSP to a single exponential decay curve (beginning from a theoretical initial value of $K \times g_{Max}$).

Tables

Table 1 E13.5 neuron model

	Length (µm)	Diameter (0) (µm)	Diameter (1) (µm)	Branch position (% Length)
Cell body	12.17	12.17		
Axon	100	2	2	
**Dend1**	87	1.8	0.75	
Dend1a	25	1.525	1	10
Dend1b	25	1.525	1	12
Dend1c	30	1.525	1	14
Dendb1d	40	1.525	1	16
**Dend2**	35	3	1.45	
Dend2a	22.62	2.6	1.45	15
Dend2b	17.44	2.6	1.45	20
Dend2c	22.62	2.6	1.45	25
Dend2d	19.6	2.6	1.45	30
**Dend3**	16.12	1.52	1.45	
**Dend4**	16.12	1.5	1.42	
**Dend5**	10.3	1.6	1.42	
**Dend6**	10	1.85	1.42	

Table 2 E17.5 neuron model

	Length (µm)	Diameter (0) (µm)	Diameter (1) (µm)	Branch position (% Length)
Cell body	17.27	17.27		
Axon	170	2	2	
Dend1	280	2.4	0.4	
Dend2	185	2.8	0.5	
Dend3	90	1.8	0.3	
Dend4	283	2.8	0.5	
Dend4a	190	1.8	0.4	5
Dend4b	150	1.8	0.4	25
Dend5	93.5	2	0.4	
Dend5a	91.9	1.8	0.4	30
Dend6	265	2.2	0.4	
Dend6a	86.9	1.8	0.4	72
Dend6b	50	1.8	0.4	80
Dend7	204	2.2	0.4	
Dend7a	147.1	1.8	0.4	40
Dend8	168.37	2.8	0.7	
Dend9	168	1.8	0.4	
Dend9a	100	1.8	0.4	10
Dend9b	133	1.8	0.4	30
Dend9c	110	1.8	0.4	70

Supplemental Experimental Procedures

Recording and staining

Because a high R_{in} of immature motoneurons may lead to erroneous values of the resting membrane potential (E_{Rest}), spike threshold (E_{Thr}) and E_{Cl}, we used equation (2) as proposed by Tyzio and collaborators, which corrects for this inaccuracy [1].

$$E'm = E^0m \times \frac{Rps - Rin}{Rps} + Eps\frac{Rin}{Rps} \Rightarrow E^0m = (E'm - Eps \times \frac{Rin}{Rps}) \times \frac{Rps}{Rps - Rin} \qquad (2)$$

where $E'm$ is the measured value, E^0m is the compensated value, Rps is the seal resistance (always ≈ 10 GΩ), Rin is the input resistance of the recorded motoneuron, and Eps is the liquid junction potential between the bath and intra-pipette solutions (12.5 mV at E13.5 and 14.3 mV at E17.5). All of the potential values were then corrected using this procedure. However, $[Cl^-]_i$, which was calculated from E_{Cl} values, was systematically below $[Cl^-]_{intra\text{-}pipette}$, thus suggesting that the potential values produced by the correction were slightly over-compensated.

An analysis of the electrophysiological data was performed off-line using pCLAMP 10.3 (Axon Instruments). The MN input resistance was determined in the current-clamp mode by injecting current pulses (duration: 1.5 s) of different intensities (I) and polarities through the recording pipette. The MN voltage (V) responses were measured, and the V/I curves were constructed. The input resistance R_{in} was measured as the slope of the linear portion of the V/I relationship. The MN capacitance c_m was collected immediately following the establishment of the whole-cell patch clamp using the Clampex Membrane Test tool. The E_{Cl} and g_{Clp} were calculated by linear fit using GraphPad Prism 6 (La Jolla, CA, USA) from current responses and collected in voltage-clamp mode to repeated brief puffs (50 ms duration) of isoguvacine (every 20 s) at different membrane potentials (Suppl. Figures 3A1-3A2). The E_{Cl} and g_{Clp} were measured before and after the series of assays designed to estimate the time course of excitatory/inhibitory effects of dGPSPs (see below). The E_{Cl} may be considered E_{GABA_AR}

because of the low HCO_3^- conductance of $GABA_AR$ in embryonic spinal MNs [2]. Consequently, the E_{Cl} was measured as the zero $I_{GABA_A}R$ current crossing voltage. The slope of this I/V curve was an estimation of the g_{Clp} (Suppl. Figure 3A2).

Electrical stimulation of the ventral funiculus

To activate local GABA-/glycinergic interneuronal projections produced on recorded E13.5 MNs, a bipolar stimulating electrode was displaced on the surface of the ipsilateral ventral funiculus (VF) rostral to the MN. Trains of 8 single pulses (1 ms duration, 10–100 μA) were applied to the VF using an ISO-Flex stimulus isolation unit (A.M.P.I., Jerusalem, Israel) driven by a programmable Master 8 Stimulator/Pulse Generator (A.M.P.I. Master-8) at various frequencies. The output spiking frequency of the E13.5 MN was then calculated at the steady-state regime (generally pulses 3 to 8). Electrical stimulations were performed in the presence of DL-2-amino-5-phosphono-pentanoic acid (AP5; 50 μM, Tocris Bioscience), 6-cyano-7-nitroquinoxaline-2,3-dion (CNQX; 20 μM, Tocris Bioscience) and dihydro-β-erythroidine hydrobromide (DHβE, 5μM, Tocris Bioscience). This pharmacological cocktail was used to block the excitatory amino acid transmissions involved in the VF inputs to spinal lumbar MNs [3] and nicotinic cholinergic transmissions, which control bursts of activity evoked after VF stimulation or occur spontaneously at early developmental stages [4]. The activation of a chloride conductance during VF stimulation was verified by measuring the reversal potential of the VF response in voltage-clamp configuration as described above, which produced values (-48.5 ± 2.5 mV, $n = 5$) not statistically different from the E_{Cl} values measured in the E13.5 MNs recorded using an E13.5-like intracellular medium (-44.3 ± 1.9 mV, $n = 6$) (non-parametric Mann-Whitney U test). The g_{Clp} value, which was calculated for VF-evoked dGPSPs, was 2.3 ± 1.1 nS ($n = 5$) and in the same range as the g_{Clp} values measured for isoguvacine-induced dGPSPs (see Results).

Stimulation and $GABA_AR$ activation

The injection of current pulses and the triggering of isoguvacine ejection were controlled with a programmable multichannel pulse generator (A.M.P.I. Master-8, Jerusalem, Israel). The threshold current pulse was adjusted to elicit 1 to 3 spikes in a series of 4 pulses that were spaced by 2 s (Suppl. Figure 3B). If this was the case (*i.e.*, the current intensity was sufficiently close to the threshold), a fifth current pulse was generated during the isoguvacine puff. If four spikes were produced, the process was aborted, and the current pulse was slightly reduced before a new series trial was performed. If no spikes occurred of the four current pulses, the process was also aborted; the current pulse intensity was then slightly increased before a new trial was performed. The series of 5 current pulses (0.5 Hz) was separated by a resting time of 20 s to avoid alterations in E_{Cl} by the overactivation of $GABA_AR$. To explore the inhibition/excitation occurring during the rising phase of the isoguvacine puff, the delay between the fifth current pulse and the onset of the isoguvacine puff was gradually modified (-20, 0, 20, 40, 60 and 80 ms) in successive assays. Each assay was repeated 4 to 10 times. The quantification of inhibition and excitation that were produced by the isoguvacine puff were then expressed by the ratio $R_{GABA.}$ This parameter represents the percentage of spikes that were triggered by depolarizing current pulses along the isoguvacine puff divided by the percentage of spikes that were triggered before the puff (at least 4 series, as illustrated in Suppl. Figure 3B3). In these experiments, we did not analyze the inhibition/excitation occurring during the falling phase of the response to isoguvacine puff because this phase was not comparable to the falling phase of a physiological synaptic event. Indeed, the time constant of this repolarizing phase (281.7 ms at E13.5 and 317.4 ms at E17.5) was much slower than the respective capacitive time course (33.7 ms and 45.9 ms) (compare Suppl. Figures 2B1-2B3 and Suppl. Figures 2C1-2C3). This difference was ascribed to the wave of isoguvacine traveling on the surface of the recorded MN and progressively reaching the $GABA_AR$ sites, even during the repolarizing phase. Therefore, this repolarizing phase was

not essentially capacitive in contrast to the falling phase of physiological GABA/glycine synaptic events (dGPSPs).

Confocal microscopy

A BX51 Olympus FluoView 500 confocal microscope (Olympus France, Rungis) was used to image neurobiotin-stained MNs. Serial optical 0.2-µm sections were obtained using an oil-immersion 60× objective with a 1.0 numerical aperture. The shrinkage was estimated from confocal images of an MN that was stained with rhodamine-dextran (0.5%; Molecular Probes) diluted in the intracellular medium before and after PFA fixation. The diameter of the MN cell body was measured in 3 dimensions. A shrinkage factor of 0.3 was estimated in the Z-axis, and corrections in this dimension were systematically applied for each reconstructed MN. Noticeable shrinkage was not observed along the X-Y axis as previously reported [5].

Computer simulations

The diameter of the neuron model cell bodies was calculated to fit the cell average body surface of actual MNs (620 μm^2 for E13.5 MN and 1249 μm^2 for E17.5 MN). The diameter of the primary and secondary dendrites was set accordingly with the corresponding canonical E13.5 and E17.5 MNs. The axon and the dendrites were constructed with a variable number of compartments depending on their length and diameter, respecting the rule diameter/length = 1/10. The axon of the E13.5 neuron model was constructed with an initial segment (length: 30 µm, diameter: 1 µm) and an axon (length: 100 µm, diameter: 1 µm). The axon of the E17.5 neuron model was constructed with an initial segment (length: 30 µm, diameter: 2 µm) and an axon (length: 170 µm, diameter: 2 µm). The axon initial segment length (AIS, 30 µm) corresponded to previously reported physiological values [6]. The properties of each compartment were independently defined.

PASSIVE PROPERTIES OF ALL OF THE COMPARTMENTS

The intra-compartmental potential, E, is described by the following differential equation:

$$\frac{dE}{dt} = \frac{I_{leak}+I_{core}+I_{ch}+I_{syn}}{cm} \qquad (3)$$

where I_{leak} is the passive leakage current and described by the following equation:

$$I_{leak} = (E_{leak} - E_m)\times G_{leak} \qquad (4)$$

and E_{leak} and G_{leak} are the equilibrium potential and the conductance of the leak current, respectively. In the present simulations, E_{leak} was used to impose an E_{Rest} on the simulated neuron. $G_{leak} = 1/R_m$; with R_m, the specific membrane resistance was set to 25,390 $\Omega\cdot cm^2$ in the E13.5 neuron model and 17,350 $\Omega.cm^2$ in the E17.5 neuron model. The resultant R_{in} was 900 MΩ and 140 MΩ in the E13.5 and E17.5 neuron models, respectively. These values correspond to the averaged R_{in} measured from actual E13.5 and E17.5 MNs. E_m is the membrane potential. I_{core} is the axial current to neighboring compartments summed over all of the neighbors:

$$I_{core} = \sum_{c\in neighbors}(E_c - E_m)\times G_{core} \qquad (5)$$

The parameter G_{core} (in S) denotes the core conductance from the compartment with respect to the neighboring compartment, as follows:

$$G_{core} = \frac{p\times diam^2}{4}\times\frac{1}{l}\times\frac{1}{R_a} \qquad (6)$$

where *diam* and l are the diameter and length of the compartment (in cm), respectively, and R_a is the specific resistance of the axoplasm (in Ω·cm). All of the computations were performed assuming a specific axoplasmic resistance, R_a, of 100 Ω·cm.

I_{ch} and I_{syn} in Equation (3) represent intrinsic and synaptic currents, respectively. The intracellular current injection can be modeled by adding the current to the compartment.

c_m is the capacitance (μF) of each compartment and was calculated as follows:

$$c_m = C_m \times area \qquad (7)$$

where *area* is the membrane surface of the compartment in cm^2 and C_m is the specific membrane capacitance (set to 1 $\mu F \cdot cm^{-2}$).

ACTIVE PROPERTIES OF THE AXON AND INITIAL SEGMENT

In addition to the passive properties, each of the axon compartments possessed active properties that were simulated by Hodgkin and Huxley (HH) Na^+ and K^+ channels. Their densities were adjusted to obtain a spike threshold (E_{Thr}) of -50.2 mV for the E13.5 and E17.5 neuron models:

$gNa_{hh}Max$ = 0.18 $S \cdot cm^{-2}$ and $gK_{hh}Max$= 0.036 $S \cdot cm^{-2}$ in the initial segment for the E13.5 neuron model

$gNa_{hh}Max$ = 0.48 $S \cdot cm^{-2}$ and $gK_{hh}Max$= 0.144 $S \cdot cm^{-2}$ in the initial segment for the E17.5 neuron model

$gNa_{hh}Max$ = 0.12 $S \cdot cm^{-2}$ and $gK_{hh}Max$= 0.036 $S \cdot cm^{-2}$ in the axon. The formalism of the HH channels was described by standard HH Na^+ and K^+ channel kinetic equations.

For Na^+ channels: $gNa_{hh} = gNa_{hh}Max \times m \times m \times m \times h$ (8)

where m = activation and h = inactivation.

Activation dynamics: $m' = \alpha_m \times (1-m) - \beta_m \times m$ (9)

with $\alpha_m = A_{\alpha m} \times (k_{\alpha m} \times (E - d_{\alpha m}))/(1 - exp(-k \times (E - d)))$ (10)

$A_{\alpha m} = 1$ (ms^{-1}); $k_{\alpha m} = 0.1$ (mV^{-1}); $d_{\alpha m} = -40$ (mV)

$\beta_m = A_{\beta m} \times exp(k_{\beta m} \times (E-d_{\beta m}))$; $A_{\beta m} = 4$ (ms^{-1}) (11)

$k_{\beta m} = -0.055556$ (mV^{-1}); $d_{\beta m} = -65$ (mV)

Inactivation dynamics: $h' = \alpha_h \times (1 - h) - \beta_h \times h$ (12)

with $\alpha_h = A_{\alpha h} \times exp(k_{\alpha h} \times (E - d_{\alpha h}))$ (13)

$A_{\alpha h}$ = 0.07 (ms^{-1}); $k_{\alpha h}$ = -0.05 (mV^{-1}); $d_{\alpha h}$ = -65 (mV)

$\beta_h = A_{\beta h}/(1 + exp(k_{\beta h} \times (E - d_{\beta h}))$ (14)

$A_{\beta h}$ = 1 (ms^{-1}); $k_{\beta h}$ = -0.1 (mV^{-1}); $d_{\beta h}$ = -35 (mV)

For K^+ channels: $gK_{hh} = gK_{hh}Max \times n \times n \times n \times n$ (15)

where n = activation.

Activation dynamics: $n' = \alpha_n \times (1 - n) - \beta_n \times n$ (16)

with $\alpha_n = A_{\alpha n} \times (k \times (E - d_{\alpha n}))/(1 - exp(-k_{\alpha n} \times (E - d_{\alpha n})))$ (17)

$A_{\alpha n}$ = 0.1 (ms^{-1}); $k_{\alpha n}$ = 0.1 (mV^{-1}); $d_{\alpha n}$ = -55 (mV)

$\beta_n = A_{\beta n} \times exp(k \times (E - d_{\beta n}))$ (18)

$A_{\beta n}$ = 0.125 (ms^{-1}); $k_{\beta n}$ = -0.0125 (mV^{-1}); $d_{\beta n}$ = -65 (mV)

Synaptic inputs

The kinetics of the PSPs were described by the alpha function conductance and defined as follows:

$i = g \times (E_m - E_{Cl})$ (19)

where i = nanoamps, g = microSiemens, and $g = 0$ for t < onset. $g = gmax \times (t - onset)/tau \times exp(-(t - onset - tau)/tau)$ (20) for t > onset

where *gmax* is the maximum conductance for the considered postsynaptic receptor channel ($gmax = g_{Clp}$). *Em* is the membrane potential, and E_{Cl} is the equilibrium potential for the ion that permeates through the channel (Cl^- ion). This value was adjusted according to the physiological measurements of E_{Cl}. The alpha function has the property that the maximum value is *gmax* and occurs at

$$t = onset + tau \qquad (21)$$

The time constant of IPSPs (*tau*) was set to 20 ms according to physiological values that were measured during the patch clamp recording experiments.

To compare the effects of the simulated dGPSPs in the E13.5 and E17.5 neuron models, we estimated the g_{Clp} that was required to evoke similar depolarizing events in both of the models (Suppl. Figure 1A) for E_{Cl} = -42 mV and E_{Rest} = -75 mV. The peak amplitude of the depolarizing response to various simulated g_{Clp} was measured in both of the models (Suppl. Figure 1A1-2), and the corresponding curves were plotted against g_{Clp} (Suppl. Figure 1A3). This analysis revealed that the g_{Clp} that was required in an E17.5 neuron model was 5.5 times larger than the g_{Clp} that was required in an E13.5 neuron model to elicit similar peak amplitudes.

The time course of g_{Cl} during a representative dGPSP as simulated in an E13.5 neuron model with g_{Clp} = 3.5 nS (Suppl. Figure 1B) indicates that g_{Clp} is reached at t = 20 ms before the dGPSP peak at t = 45 ms. To estimate the summation of the currents underlying the dGPSPs trains, the falling phase of dGPSPs was fitted with a single exponential decay that was used to estimate the conductance at t = 0 ms (Suppl. Figure 5). This onset value ($K \times g_{Max}$) was used in the equation from Le Bon-Jego et al. (2004). In these experiments, K was estimated to 2.4.

IETC MAP COLOR CODE

To accurately represent the excitatory and inhibitory effects of dGPSPs in the IETC maps, we used the following color code: 0% effects were coded in white; excitatory effects were coded in red, and inhibitory effects were coded in blue.

Excitatory effects from 0 to 10% were coded in a color gradation between white and red; excitatory effects from 10 to 20% were coded in a color gradation between red and black; and excitatory effects from 20 to 30% were coded in a color gradation between black and red. These two latter gradations were repeated several times according to the maximum effect.

The inhibitory effects from 0 to 10% were coded with a color gradation between white and blue; inhibitory effects from 10 to 20% were coded with a color gradation between blue and black; and inhibitory effects from 20 to 30% were coded with a color gradation between black and blue. These two latter gradations were repeated several times according to the maximum effect.

References

1. Tyzio R*, et al.* Membrane potential of CA3 hippocampal pyramidal cells during postnatal development. *J Neurophysiol* **90**, 2964-2972 (2003).

2. Bormann J, Hamill OP, Sakmann B. Mechanism of anion permeation through channels gated by glycine and gamma-aminobutyric acid in mouse cultured spinal neurones. *J Physiol* **385**, 243-286 (1987).

3. Brocard F, Vinay L, Clarac F. Gradual development of the ventral funiculus input to lumbar motoneurons in the neonatal rat. *Neuroscience* **90**, 1543-1554 (1999).

4. Czarnecki A*, et al.* Acetylcholine controls GABA-, glutamate-, and glycine-dependent giant depolarizing potentials that govern spontaneous motoneuron activity at the onset of synaptogenesis in the mouse embryonic spinal cord. *J Neurosci* **34**, 6389-6404 (2014).

5. Li Y, Brewer D, Burke RE, Ascoli GA. Developmental changes in spinal motoneuron dendrites in neonatal mice. *J Comp Neurol* **483**, 304-317 (2005).

6. Le Bras B*, et al.* In vivo assembly of the axon initial segment in motor neurons. *Brain Struct Funct* **219**, 1433-1450 (2014).
